# The relationship between lay beliefs about the world and pandemic-related beliefs, attitudes, and behaviors

**DOI:** 10.1371/journal.pone.0338367

**Published:** 2025-12-10

**Authors:** Julia Marie Jankowski, Kata Sik, Veronika Job

**Affiliations:** Department of Occupational, Economic and Social Psychology, University of Vienna, Vienna, Austria; University of Padova, ITALY

## Abstract

The Covid-19 pandemic created a strong need to understand how people can be motivated to engage in protective health behaviors (such as vaccination). Past research suggests that perceiving a health threat as serious and protective behaviors as beneficial increases people’s motivation to engage in protective behavior. However, perceptions of the seriousness of the threat and the effectiveness of protective behavior can be ambiguous in the context of a novel, global pandemic. Research on climate change suggests that people’s core understanding of the world (as either stable and unchangeable or dynamic and influenced by humans) influence whether people perceive such abstract threats to be serious and protective behaviors to be effective on a large scale. Across five online studies (*N *= 1663), we investigated the correlational relationship and causal effect of these lay beliefs about the world on beliefs, attitudes, and behavioral inclinations regarding both Covid-19 and a hypothetical pandemic scenario. The results did not support a causal effect of lay beliefs about the world, but consistently showed correlational relationships. A sixth online study (*N* = 410) tested a possible reversed causal effect of pandemic-related behavior inclinations being justified by shifting one’s lay beliefs about the world accordingly. However, we did not find evidence for this effect either. These results indicate that lay beliefs about the world are associated with perceptions of pandemic health threats, but their causal role is unclear.

## Introduction

The outbreak of the Covid-19 pandemic has put extreme demands on medical systems, intensive care units, and global health [1,2]. According to the WHO, the coronavirus has infected over 774 million people and caused over 7 million people’s death as of February 2024 [3]. Research suggests a high probability of observing similar pandemics in the coming decades [4]. Thus, there is a clear need to understand how to motivate people to take steps to mitigate such pandemics. There are several ways through which people can influence the course of a pandemic– e.g., by following rules and regulations that are implemented to “flatten the curve” of the pandemic or by getting vaccinated to achieve herd immunity [5]. However, the Covid-19 pandemic showed that several people would not follow the regulations, refrain from getting vaccinated or even deny the pandemic threat altogether [6–8]. Thus, it is vital to understand the factors that motivate people or prevent them from engaging in behavior that helps the fight against pandemics such as Covid-19. In this paper, we are interested in how people’s core understanding of the world (i.e., whether they believe that the world at a large scale is changeable and specifically shaped by humans or not) influences people’s beliefs, attitudes and behaviors related to a surging pandemic. Believing that the world is rather stable and unchangeable might lead people to underestimate the threats of a new situation such as the Covid-19 pandemic. Furthermore, believing that humans have little impact on the world at large might additionally lead people to feel like their protective behavior is ineffective. Both would likely discourage people from engaging in protective behaviors.

### Health-related behavior

One of the most frequently used frameworks to understand health and illness behaviors is the Health Belief Model [9]. The Health Belief Model states that individuals’ beliefs along with cues to action influence their health behavior. The beliefs include perceptions of susceptibility to and severity of the disease, benefits of protective behaviors, barriers to action, and self-efficacy. Multiple studies have supported the validity of the model in the context of the Covid-19 pandemic regarding protective behavior [6,10–13]. Perceived benefits have often emerged as one of the strongest among the predictors of both vaccine acceptance [12,13] and following governmental regulations and taking health precautions [6]. Still, higher perceived severity, self-efficacy, and cues to action have also been connected to higher intentions to get vaccinated against Covid-19 [12,13] and perceived severity and self-efficacy are also related to more engagement in preventive behaviors [6,11]. Meanwhile, higher perceived barriers to action have been connected to lower vaccination intention [12,13] and less preventive behavior [11]. Thus, the Health Belief Model is an adequate framework to study Covid-19 related behavior.

We use the Health Belief Model as a guiding framework; however, we only analyze two components of it, namely perceived severity and perceived benefits. We focus specifically on these two because the global nature of the Covid-19 pandemic has notable implications for both of them. First, Covid-19 presented a rather sudden, but distant and abstract threat to people. Although the whole world was affected, most people did not have immediate contact with severe cases of Covid-19. Additionally, the severity of a pandemic disease is unclear at the beginning even from a scientific point of view, and reliable information needs to be gathered over time. This may make its perceived severity harder to grasp, leaving room for individuals’ own interpretation. Second, perceived benefits can include benefits for the individual (e.g., protecting oneself from getting infected), but also benefits for others or the general society (e.g., protecting more vulnerable people or contributing to herd immunity by getting vaccinated). Even though previous studies mostly focused on individual benefits (e.g., 6), the benefits to others and society (i.e., perceived benefits at the collective level) might be especially important in a large health crisis like a pandemic. In a pandemic, one’s own actions do not only impact one’s own health but also have implications for society-wide outcomes such as the health of others and the general course of the pandemic. Such social benefits of vaccination and mitigation efforts might thus be as important motivators as individual benefits. This emphasis reflects a referent shift within the Health Belief Model’s facet perceived benefits (from self to others and society). Importantly, social benefits are independent of one’s personal susceptibility to the disease. For many people (i.e., young, and healthy ones), the individual threat of Covid-19 and thus the individual benefit of protective behavior was rather low (e.g., the infection fatality rate was below 0.1% for people under 39 years [14]). These people might rather be motivated by the social benefits (i.e., protecting vulnerable groups or mitigating the pandemic in general). Thus, because a pandemic context may have unique implications for perceptions of the severity of the disease and the benefits of preventive measures, this paper will focus on these two aspects. Our aim is to clarify the impact of these two Health Belief Model components in the pandemic context, focusing on the collective benefits rather than on individual benefits.

Overall, increasing the perception of a pandemic (e.g., Covid-19) as serious and the belief in the efficacy of one’s behavior to not only protect oneself but also alleviate the strain on society seems to be a viable way to increase pandemic-related protective behavior. In our studies, we thus focused on how people’s core understanding of the world affected the perception of the seriousness of a pandemic (such as Covid-19) and the efficacy of mitigation efforts and hence protective behavior.

### Insights from climate change research

Both factors – belief in the seriousness of the issue and belief in the efficacy of one’s own behavior – are key factors in climate change mitigation. Indeed, both issues share common characteristics. Just like climate change, a pandemic represents a rather abstract, distant threat. People do not usually perceive the negative effects of climate change in their daily lives, just like most people did not experience the severe health consequences of Covid-19 firsthand during the pandemic. In the US, there have been about 460,000 hospitalizations (as of June 2, 2023) corresponding to ca. 0.1% of the population, so most people did not personally know one of them [15]. As with climate change, the threat of Covid-19 was mostly conveyed in the news. This made it seem far away and not personally relevant, making it easy to ignore [16]. Additionally, in both issues, collective action is needed. Climate change and a global pandemic can both only be addressed if a substantial number of people engage in protective behavior. Thus, one’s personal behavior can seem irrelevant on the broad scale. Both – the abstract threat and unclear impact of the individual – might reduce the perceived relevance of protective behavior, especially since it is also connected to negative, short-term consequences (e.g., not seeing one’s family, wearing an annoying mask).

Climate change research provides us with some insights on how to still motivate protective behavior. First, it indicates that educating people and trying to convince them with scientific information has limited effects. Knowledge about climate change is only weakly connected to climate change belief [17]. Furthermore, confronting skeptic people with facts is often met with reactance [18]. A similar dynamic could be observed regarding Covid-19: providing facts about Covid-19 and protective measures did not seem to be effective [19]. Some researchers argue that instead, underlying worldviews and interests are crucial as they shape how people interpret facts around climate change [20]. This might as well be true for perceptions about a pandemic such as Covid-19.

### Lay beliefs about the world

An underlying worldview that has been identified to influence beliefs and behavior regarding climate change are lay beliefs about the world. Lay beliefs about the world concern the belief as to whether the world has a “fundamental, uncontrollable nature [or whether] people, through their efforts, can shape society and its institutions” [21]. An entity belief about the world refers to the conviction that the world and its institutions are fundamentally immutable and uncontrollable (although transient societal trends might exist temporarily) [21]. An incremental belief about the world refers to the belief that people have an impact on the world and its institutions and can change them [21]. These beliefs fundamentally shape one’s perception of and relation to the world. For people with an entity belief, the world is fixed. Such a view would motivate to accept the world as it is and adjust to it. For people with an incremental belief, the world is malleable. This should motivate them to change the world actively where they feel the need.

Lay beliefs about the world have been shown to be related to distinct beliefs, emotions and behavioral patterns regarding climate change and other social issues. Incremental theorists believe more strongly that climate change is happening, man-made and a relevant threat [22,23]. Additionally, incremental theorists have more favorable beliefs and emotions about the solvability of social issues. They have stronger efficacy beliefs, i.e., they believe that their own actions can contribute to mitigating climate change [22,23]. They also feel more hopeful that social injustices and conflicts will be solved peacefully [24,25]. Finally, the differences in efficacy belief and hope promote distinct behavioral patterns to target the problem. Incremental theorists engage in more collective action (e.g., demonstrations) and engage in more behavior to target the problem (e.g., more pro-environmental behavior in the context of climate change) [22,23,25]. Entity theorists instead turn to more non-normative action (i.e., violent behavior) [25].

Because climate change and global pandemics (e.g., Covid-19) share core features, we assume that lay beliefs about the world also play a role in the perception of pandemics. Pandemics entail a fundamental change in people’s social world (e.g., actions that were previously normal are suddenly considered risky, and a previously safe environment now holds an invisible threat). Lay beliefs about the world might matter in whether one acknowledges or is skeptical about this drastic, global change. Since the rise of the pandemic, some people have downplayed the risk of Covid-19, despite clear evidence of the virus being serious [8]. An entity belief about the world might have contributed to that, since believing in a fixed world is at odds with this drastic change. Furthermore, entity theorists might not believe that their personal behavior is relevant to combat the pandemic. According to the Health Belief Model, these differences in perceived severity and benefits should lead to differences in behavior. Entity theorists might, thus, not engage as much in behaviors to combat the pandemic (e.g., wearing masks, getting vaccinated) as their incremental counterparts. Likewise, entity theorists should also see restrictions to combat Covid-19 (e.g., closing shops) as less effective and thus disapprove of them.

Out of several important behavioral tendencies and intentions regarding Covid-19, one of the most important aspects was people’s willingness to get vaccinated. By getting vaccinated, one cannot only protect oneself, but also other people by decreasing virus transmission and the risk of new mutations to arise. In 2019, the World Health Organization (WHO) announced vaccine hesitancy as one of the ten threats to global health [26]. Future pandemic situations might face similar vaccine hesitancy as did the Covid-19 pandemic [27]. Therefore, vaccine willingness was a key dependent variable in our studies (next to other behavioral intentions and attitudes).

### Present research

The present research was conducted from March 2021 to August 2023 as a sequential program of studies designed to progressively test the relationship between lay beliefs about the world and pandemic-related beliefs, attitudes, and behaviors. Study 1 provided an initial correlational test, examining the associations of lay beliefs about the world with Covid-19-related beliefs, attitudes, and behaviors. Building on this, Study 2 moved beyond correlation by experimentally manipulating lay beliefs about the world to test their causal effect on Covid-19-related outcomes. As the effects in Study 2 did not match the results of Study 1, in Study 3a, we introduced a hypothetical, novel pandemic scenario. This allowed us to investigate the possibility that participants’ ongoing prior experiences with Covid-19 masked the causal effect and to test the generalizability to contexts beyond Covid-19. Studies 3b-c, then expanded Study 3a by strengthening the manipulation (3b) and increasing the sample size (3c) to improve statistical power. Finally, Study 4 tested the reversed causal direction by asking participants to imagine either adhering to or failing to adhere to protective measures and then assessing whether their lay beliefs about the world shifted in line with that behavior. This allowed us to investigate whether people would adjust their lay beliefs about the world to justify their behavior. Together, this progression allowed us to systematically test the relationship between lay beliefs about the world and pandemic-related outcomes across correlational and causal designs, as well as across real and hypothetical pandemic contexts.

All studies were approved by the Departmental Review Board of *[university name blinded for review]* (project numbers: 2021/M/003, 2021/M/010, 2022/M/002, 2023/M/005). Studies 2, 3a-c and 4 were pre-registered (https://aspredicted.org/L5Z_RS8, https://aspredicted.org/F7X_3Q2, https://aspredicted.org/2QL_QFG, https://aspredicted.org/HKF_RCD, https://aspredicted.org/9B4_X2B).

## Study 1

Study 1 explored the association of lay beliefs about the world with Covid-19-related beliefs, attitudes, and behaviors. We focused on Covid-19-related behavior change, willingness to get vaccinated against Covid-19, persistence behavior to support a Covid-19 relief fund, attitudes towards Covid-19 measures, perceived severity of the disease (Covid-19 belief) and perceived benefits of mitigation efforts (Covid-19 efficacy belief).

### Method

#### Participants.

208 US Americans completed an online study via Amazon Mechanical Turk (recruiting period: 12/03/2021–31/03/2021). After excluding participants that failed an attention check (*N* = 22) or experienced technical problems (*N* = 5), the final sample size was *N* = 181 (51% female, 48% male, 1% non-binary; mean age 42 years, *SD* = 12.3). Most participants (78%) were White; the others were Asian (9%), Black (6%), Hispanic (4%), Native American (1%) or indicated another ethnicity (2%). Overall, participants slightly tended to be politically liberal (*M* = 2.61, *SD* = 1.28 on a scale from *Very liberal* [1] to *Very conservative* [5]).

#### Procedure and measures.

After participants gave written consent to the participation information and the data protection form, they completed different questionnaires (e.g., on their lay beliefs about the world and personality), Covid-19-related measures, and participated in a persistence task. Lastly, they completed some general demographic questions, and they were thanked and debriefed. The main variables of interest will be described in the following sections (the full material is available under the following link: https://osf.io/57rpw/?view_only=2ef554e052b94b4b9335d1cdf1eafbcb).

**Lay beliefs about the world.** The Lay Beliefs about the World Scale [28] included 3 items (e.g., “Our world has its basic or ingrained dispositions, and you really can’t do much to change them”) and participants responded on a 6-point Likert scale from *Strongly disagree* [1] to *Strongly agree* [6]. Responses to all items were averaged (α = 0.93), higher scores indicate a stronger entity belief about the world.

**Covid-19-related behavior change.** Participants reported on their behavior over the past seven days regarding nine Covid-19 guidelines (e.g., “Wash your hands regularly with soap and water for at least 20 seconds or clean them with alcohol-based hand rub”). Participants indicated how different their behavior regarding these guidelines had been relative to before the pandemic on a 4-point Likert scale from *Exactly the same* [1] to *Very different* [4]. Responses to all guidelines were averaged, with higher scores reflecting larger behavior change to conform with Covid-19 guidelines. To assess the internal consistency (α = 0.81), one item (“Stay home if you feel unwell”) was removed, as most participants had missing data (because they had stated that they had not been unwell in the past seven days).

**Vaccination intention.** Intention to get vaccinated against Covid-19 was measured by two questions. The first one asked whether participants had already registered or tried to get the Covid-19 vaccine (*yes* or *no*). If participants hadn’t done so yet, they were asked if they would get vaccinated (once a vaccine was approved by the FDA and available) (*yes* or *no*). The answers on both questions were summarized as *yes* if any of the two questions were answered with *yes* and as *no* if both questions were answered with *no*.

**1-back persistence task.** Participants completed a very easy, rather boring N(1)-back letter memory task [29]. They were told that they could complete as many trials as they wanted (i.e., they could quit the task and proceed to the next study part whenever they wanted). They were informed that the number of correctly solved trials would translate into a donation to a Covid-19 relief fund (specifically the nonprofit, nonpartisan organization Direct Relief). A few sentences described the organization’s mission and measures. It was clarified that participants’ personal compensation was not dependent on their performance on this task (however, they were also not paid extra for the time they spent on the task). We used the number of correctly solved items as an index of persistence. Higher values reflect longer persistence on the task.

**Covid-19 threat belief.** Perceived threat of Covid-19 was measured with a 10-item scale (e.g., “Covid-19 is a real threat”, “Negative consequences of Covid-19 are minor”) similar to an existing questionnaire on the pandemic swine flu [30]. Participants responded on a Likert scale from *Strongly disagree* [1] to *Strongly agree* [6]. Responses to all items were averaged (α = 0.96), with higher scores representing stronger belief in the pandemic being a real threat.

**Covid-19 efficacy belief.** For each Covid-19 guideline used to assess Covid-19 related behavior change, participants indicated their efficacy belief regarding that guideline (“I feel that by adhering to this guideline, I can make a real difference in the fight against the pandemic.”). Participants responded on a 5-point Likert scale from *Strongly disagree* [1] to *Strongly agree* [5]. Responses on all guidelines were averaged, with higher scores reflecting higher efficacy beliefs regarding Covid-19 protective behavior. To assess the internal consistency (α = 0.95), again one item (“Stay home if you feel unwell”) was removed as most participants had missing data (because they had not been unwell in the past seven days).

**Covid-19 attitudes.** In order to assess attitudes towards Covid-19 measures, we used a scale on the support of specific governmental measures (e.g., “Closing daycares, schools and universities”) [31]. Participants responded how much they agreed with each initiative on a 6-point Likert scale from *Strongly disagree* [1] to *Strongly agree* [6]. Responses to all items were averaged (α = 0.94), with higher scores indicating stronger support for the measures.

**Political ideology.** Participants were asked to indicate their political ideology (“In general, do you think of yourself as…”) on a scale from *Very liberal* [1] to *Very conservative* [5].

### Results

Lay beliefs about the world were associated with all Covid-19-related outcomes (see Table 1). First, lay beliefs about the world were related to Covid-19-related behavior change and intention. Participants holding more of an entity belief changed their behavior less to follow Covid-19 guidelines (*r*_*Pearson*_ = −.20) and were less willing to get vaccinated (*r*_*Point-biserial*_ = −.28). To assess the relationship with persistence on the N-back task, we conducted a negative binomial regression to account for zero-inflation in the data. It showed that holding an entity belief was related to less persistence on the task, *IRR* = 0.78, *CI*[0.60, 1.01], *p* = .036. Specifically, a shift of one standard deviation towards an entity belief was associated to a 22% decrease in solved trials. Second, lay beliefs about the world were related to beliefs regarding Covid-19. Namely, participants holding an entity belief believed to a lesser extent that Covid-19 is a real threat (*r*_*Pearson*_ = −.25), and that their behavior could make a difference regarding the pandemic (*r*_*Pearson*_ = −.26). Third, lay beliefs about the world were related to attitudes regarding Covid-19 measures. Specifically, participants holding an entity belief were less supportive of governmental measures to combat Covid-19 (*r*_*Pearson*_ = −.21).

**Table 1:** Correlation Matrix of the Main Variables in Study 1.

Variable	*M*	*SD*	α	1	2	3	4	5	6	7
1. Entity world belief	3.70	1.30	.93							
2. Covid-19 related behavior change	2.72	0.59	.81	−.20**						
3. Vaccination intention^1^	0.75	0.44		−.28**	.41**					
4. N-back persistence for donation^2^	71.82	132.75		−.11	−.03	.12				
5. Covid-19 threat belief	5.09	1.14	.96	−.25***	.39***	.65***	.07			
6. Covid-19 efficacy belief	4.09	0.99	.95	−.26***	.40***	.53***	−.04	.79***		
7. Attitude towards Covid-19 related measures	3.82	1.28	.94	−.21**	.29***	.38***	−.04	.70***	.68***	
8. Political ideology	2.61	1.28		.24**	−.15*	−.31***	.07	−.49***	−.36***	−.40***

*N* = 181. Pearson correlations were conducted unless otherwise indicated. Higher values of political ideology represent a stronger conservative (vs. liberal) view. ^1^Point-biserial correlations due to vaccination intention being binary. ^2^N = 150 due to a technical error in the data collection of *n* = 31 participants. * *p* < .05, ** *p* < .01, *** *p* < .001.

Past studies have shown that lay beliefs about the world are related to political ideology, with conservatives holding more of an entity belief than liberals [22–24] and the same emerged in our study (see Table 1). Since political ideology was also associated with the Covid-19 related outcomes (see Table 1), we conducted multiple regression analyses for each outcome, including political ideology as a control variable. Table 2 shows that the same patterns hold when political ideology is included as a control variable. Thus, lay beliefs about the world seem to be connected to the Covid-19 related outcomes independently of political ideology.

**Table 2:** Results of Multiple Regression Analyses of Relationship of Lay Beliefs About the World with Covid-19 Related Behavior, Beliefs, and Attitudes Controlling for Political Ideology.

Outcome variable	Estimate (β, *OR,* or *IRR*)	*df*	*t* / *z*	*p*
Covid-19 related behavior change	−0.17	178	−2.30	.022*
Vaccination intention^1^	0.64	178	−2.22	.027*
N-back performance for donation^2^	0.75	147	−2.33	.020*
Covid-19 threat belief	−0.14	178	−2.18	.031*
Covid-19 efficacy belief	−0.18	178	−2.55	.012*
Attitude towards Covid-19 related measures	−0.12	178	−1.74	.084^†^

*N* = 181. Linear regressions were conducted and standardized β reported unless otherwise indicated. Higher values of lay beliefs about the world represent a stronger entity belief. ^1^Binomial regression due to binary outcome variable. Odds Ratio is reported. ^2^*N* = 150 due to a technical error in the data collection of *n* = 31 participants; Negative binomial regression due to count data with a zero-inflation; Incidence Rate Ratio is reported. * *p* < .05, ^†^ *p* < .1.

### Discussion

In the first study, we found consistent associations between lay beliefs about the world and Covid-19 related behaviors, beliefs, and attitudes. First, participants with an incremental belief – i.e., the belief that the world is changeable – showed more behavior inclinations to mitigate Covid-19. They persisted longer on a boring task to financially support a Covid-19 relief fund. They also changed their behavior more to follow Covid-19 guidelines and reported higher willingness to get vaccinated. Second, a higher incremental belief was related to higher threat belief and higher efficacy beliefs about their mitigation efforts. Last, people with more of an incremental belief also showed more positive attitudes towards Covid-19 rules and regulations to support the mitigation of the pandemic. Thus, lay beliefs of the world seem to be associated with Covid-19 related beliefs, attitudes, and behaviors.

## Study 2

The aim of study 2 was to explore the causal effect of lay beliefs about the world. We focused on willingness to get vaccinated against Covid-19 among people who had not yet been vaccinated as the main outcome variable. Getting vaccinated was an essential step to mitigate the Covid-19 pandemic [27]. We expected that those who would be exposed to the incremental belief about the world condition, would be more willing to get vaccinated than those who were exposed to the entity belief about the world condition. In addition to the manipulation’s effect on vaccination willingness, in further exploratory analyses, we assessed the effects on beliefs in the threat of Covid-19 and the efficacy of protective behaviors as well as Covid-19 policy support.

### Method

#### Participants.

A power analysis determined that we need 118 participants to obtain a power of 80% with a standard significance level of α = 0.05 to detect the effect on vaccination willingness from a pilot study (more detailed explanation of the sample size rationale is presented in the preregistration: https://aspredicted.org/L5Z_RS8). The data collection was conducted 25/06/2021–26/06/2021 via Amazon Mechanical Turk. 478 participants from the US completed the study. 80 participants were excluded because they failed an attention check, and another participant was excluded because they indicated that their data should not be used. Thus, 397 participants were included in the secondary analyses (58% female, 41% male, 1% non-binary; mean age 43 years, *SD* = 13). Participants were mostly White (87%) with others being Black (5%), Asian (4%), Hispanic (2%) or another ethnicity (2%). The sample slightly tended to be politically liberal (*M* = 2.71, *SD* = 1.22 on a scale from *Very liberal* [1] to *Very conservative* [5]). The sample was evenly distributed across the randomly assigned experimental groups (*N*_incremental_ = 204, *N*_entity_ = 193).

To test our main hypothesis, we only included people who were not vaccinated yet. This was 29% of the overall sample (*N* = 114; 61% female, 39% male; mean age 40 years, *SD* = 11.2). Again, most participants were White (88%) with others being Black (6%), Hispanic (3%), Asian (1%), Native American (1%) or another ethnicity (2%). This subsample slightly tended to be politically conservative (*M* = 3.37, *SD* = 1.23 on a scale from *Very liberal* [1] to *Very conservative* [5]). The subsample was also evenly spread across the incremental belief (*N* = 60) and entity belief group (*N* = 54).

#### Procedure and measures.

After participants gave written consent to the participation information and the data protection form, they read a passage about whether the world was rather dynamic or fixed (lay beliefs about the world manipulation) and answered questions on the text. Following this, participants reported their vaccination status and vaccination intention in case they were not vaccinated yet. Subsequently, participants completed further measures on Covid-19 related beliefs and attitudes, a manipulation check, and additional questionnaires and demographic questions (the full material can be found under https://osf.io/57rpw/?view_only=2ef554e052b94b4b9335d1cdf1eafbcb).

**Lay beliefs about the world manipulation.** Participants read a short article about whether the world around them is known to be fixed or malleable based on scientific evidence [24]. After the manipulation, we presented four reading check questions to ensure that the participants read and understood the article. If people failed any of these items, they could read the text again once. If they gave a wrong answer again, they could not participate in the study.

**Manipulation check.** The same lay beliefs about the world scale (α = 0.94) was used as in Study 1 to confirm whether the manipulation had worked.

**Vaccination intention.** Participants who had indicated that they had not been vaccinated yet (neither completely nor the first shot) answered one item on their vaccination intention (“Do you intend to get vaccinated against Covid-19 (once it is available to you)?”, *yes* or *no*).

**Covid-19 belief.** The same Covid-19 belief scale was used as in Study 1 (α = 0.95).

**Covid-19 efficacy belief.** As the efficacy belief measure in the Study 1 was rather long and repetitive, with similar responses across all guidelines, we used a shorter measure in Study 2. We adapted a scale on beliefs about the effectiveness of sustainable behavior [23] to the Covid-19 context. The final scale included 5 items on the efficacy of protective behavior in general (e.g., “I feel like any action I take to mitigate the Covid-19 pandemic is only a ‘drop in the bucket’ and won’t make a difference.”). Participants responded on a 6-point Likert scale from *Strongly disagree* [1] to *Strongly agree* [6]. An additional item focused specifically on the perceived efficacy of getting vaccinated (“I feel that by getting vaccinated, I can make a real difference in the fight against the pandemic.”). People responded on a 5-point Likert scale from *Strongly disagree* [1] to *Strongly agree* [5]. This last item was rescaled before aggregating the 6 items (α = 0.90). Higher scores on this measure reflect higher efficacy beliefs.

**Covid-19 policy support.** We adapted a measure on attitudes towards refugee support [32] to assess attitudes towards a policy to provide vaccines to poorer countries. Participants read a short description of a recent proposal by the WHO to mitigate the pandemic (namely, the necessity of providing 11 billion Covid-19 vaccine doses to poorer countries). We further informed participants that the US had already announced a policy to provide 500 million vaccine doses, but the country could further support this case. Then, we asked two questions on participants’ attitudes of the US providing further support (e.g., “How much do you agree that the US should further support vaccination in poorer countries beyond the agreed 500 million vaccine doses?”). Participants responded to these questions on a 6-point Likert scale from *Strongly disagree* [1] to *Strongly agree* [6]. We aggregated these scores (α = 0.97) in a way that higher scores reflect stronger support towards providing vaccines to poorer countries. Two additional exploratory items were assessed (see supplement).

#### Statistical analysis.

We performed the pre-registered statistical analyses with R 4.3.2 [33]. First, we used a *t*-test to confirm that the manipulation had worked. Then, we conducted a logistic regression to investigate the effect of the lay beliefs about the world manipulation on vaccination intention. Additionally, we conducted *t*-tests to investigate the effect of the lay beliefs about the world manipulation on the other Covid-19 related outcomes.

### Results

#### Preliminary results.

Overall, participants in the entity belief group reported a significantly stronger entity belief (*M* = 3.59, *SD* = 1.28) than participants in the incremental belief group (*M* = 2.97, *SD* = 1.26). The effect was of medium size, *t*(395) = −4.85, *p* < .001, *d* = −0.49. Among non-vaccinated participants, those in the entity belief group reported a stronger entity belief (*M* = 3.87, *SD* = 1.32) than non-vaccinated participants in the incremental belief group (*M* = 3.41, *SD* = 1.22). However, the difference was only marginally significant and the effect was small, *t*(112) = −1.94, *p* = .055, *d* = −0.36. Besides, *t*he groups did not differ in gender, ethnicity, age, or political orientation neither when looking at the whole sample (*p* > .24) nor at the non-vaccinated participants only (*p* > .068).

All Covid-19 related variables were significantly correlated among each other (Table 3). Besides, lay beliefs about the world related to all Covid-19 related variables: a stronger entity belief about the world correlated with lower vaccination intention, Covid-19 threat and efficacy beliefs, Covid-19 policy support, and lower likelihood of being vaccinated (Table 3).

**Table 3:** Correlation Matrix of the Main Variables in Study 2.

Variable	*M*	*SD*	α	1	2	3	4	5	6
1. Entity world belief	3.27	1.31	.94						
2. Vaccination intention^1^	0.19	0.40	–	−.27**					
3. Covid-19 threat belief	4.82	1.21	.95	−.30**	.73**				
4. Covid-19 efficacy belief	4.33	1.24	.90	−.29**	.69**	.78**			
5. Covid-19 policy support	4.21	1.64	.97	−.26**	.54**	.63**	.65**		
6. Political ideology	2.71	1.22	–	.27**	−.52**	−.55**	−.45**	−.49**	
7. Vaccination status^2^	0.71	0.45	–	−.23**	–	.75**	.78**	.63**	−.45**

*N* = 397. Pearson correlations were conducted unless otherwise indicated. Entity World Belief refers to participants’ responses on the manipulation check. Higher values of political ideology represent a stronger conservative (vs. liberal) view. ^1^*N* = 114 (only non-vaccinated participants); point-biserial correlations due to vaccination intention being binary. ^2^Point-biserial correlations due to vaccination status being binary (0 = non-vaccinated, 1 = (partially) vaccinated). ** *p* < .01.

#### Covid-19-related outcomes.

Contrary to our prediction, there was no significant difference between the two experimental groups with regard to the intention to get vaccinated against Covid-19 (incremental belief group: 21.6%; entity belief group: 16.6%), *OR* = 0.72, *CI*[0.27, 1.84], *p* = .500.

There were no differences between the two experimental groups regarding any of the other Covid-19 related variables either (Table 4).

**Table 4:** Tests of Differences Between the Experimental Groups Regarding the Covid-19 Related Outcomes.

Outcome variable	*M* (*SD*)		*df*	*t*	*p*	*d*
	Incremental	Entity				
Covid-19 threat belief	4.79 (1.22)	4.85 (1.19)	394.6	−0.47	.641	−0.05
Covid-19 efficacy belief	4.29 (1.25)	4.38 (1.22)	394.7	−0.71	.479	−0.07
Covid-19 policy support	4.21 (1.52)	4.21 (1.77)	379.7	−0.01	.993	−0.00

*N* = 397. Two-sided t-tests were conducted.

### Discussion

Contrary to the predictions, Study 2 did not find a causal effect of the manipulation of lay beliefs of the world on vaccine intention or any other Covid-19 related outcomes. The manipulation successfully changed people’s lay beliefs about the world (albeit only marginally significantly among non-vaccinated participants), but these changes did not translate to changes in the outcomes. However, as in Study 1, we found correlational relationships between lay beliefs about the world and Covid-19 related outcomes.

A possible explanation for these results is that beliefs and attitudes around Covid-19 were already very fixed at the time of Study 2 and not easily changeable anymore. Starting with the surge of Covid-19 at the beginning of 2020, people’s beliefs, attitudes, and behavior inclinations regarding Covid-19 might have formed under the influence of their natural lay beliefs about the world. Thus, we observe correlational relationships in both Study 1 and 2. However, over time the beliefs, attitudes, and behavior inclinations regarding Covid-19 might have become fixed due to intense debates with hardened fronts and a general polarization of the topic. At this point, these outcomes might not have been susceptible to changes in lay beliefs about the world anymore. This might have been the reason Study 2 (conducted in June 2021) did not find an effect of the lay belief about the world manipulation.

This suggests that while lay beliefs about the world manipulation might not affect Covid-19 related perceptions anymore, it might well influence the formation of beliefs, attitudes, and behavioral inclinations regarding a new issue (where people do not have fixed perceptions yet). Therefore, we used a hypothetical pandemic scenario for Studies 3a-c.

## Study 3a

The aim of Studies 3a-c was to test if lay beliefs about the world would influence the formation of beliefs, attitudes, and behaviors about a novel pandemic threat that is not Covid-19. We created a pandemic scenario where – similar to Covid-19 – people’s personal behavior contributes to an overall solution. To ensure transferability to Covid-19, the proposed measures in this scenario were similar (e.g., vaccination and personal behavior change) while the specific details were different (e.g., different name, different symptoms and transmission and some unique measures). We expected that those participants who would be exposed to the incremental belief about the world manipulation, would have [1] a stronger belief in the threat; [2] stronger belief in the efficacy of mitigation efforts; [3] more supportive attitudes towards governmental measures against the pandemic; and [4] stronger behavioral intention concerning mitigation behavior.

### Method

#### Participants.

The study was pre-registered at the following link: https://aspredicted.org/F7X_3Q2. A power analysis determined a sample of 278 participants to obtain a power of 80% with a standard significance level of α = 0.05 to detect an effect of *d* = 0.3 on a one-sided *t*-test. We recruited 308 people from the US via Prolific (recruiting period: 16/02/2022–03/03/2022) to accommodate exclusions when people failed an attention check (*N* = 25), a reading comprehension question (*N* = 6) or indicated that their data should not be used (self-exclusion; *N* = 6). We analyzed 271 participants’ data (49% female, 49% male, 1% non-binary; mean age 37 years, *SD* = 13.8). Participants were mostly White (61%) with others being Asian (17%), Hispanic (12%), Black (9%) or another ethnicity (1%). The sample slightly tended to be politically liberal (*M* = 2.4 (*SD* = 1.1) on a scale from *Very liberal* [1] to *Very conservative* [5]). The sample was evenly distributed across the randomly assigned experimental groups (*N*_incremental_ = 141, *N*_entity_ = 130).

#### Procedure and measures.

After participants gave written consent to the participation information and the data protection form, they read a passage about whether the world was rather dynamic or fixed (lay beliefs about the world manipulation). Then, they read the first part of the hypothetical pandemic scenario and answered questions regarding the perceived threat of this pandemic, their intended behavioral changes, their personal and collective efficacy beliefs regarding the situation, and their attitudes towards measures to slow down the spread of the virus. Next, they read the second part of the scenario and answered further questions on the perceived efficacy of a vaccine, their vaccination readiness, and vaccination intention within the scenario. Finally, participants answered some control questions on the comparability of the scenario and Covid-19.

Below, we explain the measures of interest to this paper, the full material can be found under https://osf.io/57rpw/?view_only=2ef554e052b94b4b9335d1cdf1eafbcb. Descriptive statistics of the main variables are reported in Table 5. Descriptive statistics of the control questions are in the supplement. The experimental manipulation and manipulation check were the same as in Study 2. Regarding the dependent measures, scales from Study 1 and Study 2 were adapted to fit the novel pandemic scenario. Six items of the Covid-19 threat belief scale, eight items from the Covid-19 related behavior change scale and the full Covid-19 attitude scale from Study 1 were adapted to focus on the novel pandemic instead. Likewise, the five items of the Covid-19 efficacy belief scale from Study 2 that focused on the efficacy of protective measures in general were adapted to focus on the novel pandemic scenario. Furthermore, the item on vaccination intention from Study 2 was adapted to allow for a more nuanced response (from *Very unlikely* [1] to *Very likely* [5] instead of only *Yes* or *No*). Additionally, we included two new scales on vaccine readiness and efficacy beliefs specifically about vaccination.

**Table 5:** Correlation Matrix of the Main Variables in Study 3a.

Variable	*M*	*SD*	α	1	2	3	4	5	6	7	7a	7b	8	8a	8b
1. Entity World Belief	3.51	1.26	.91												
2. Vaccination Intention	4.32	1.15	–	−.30**											
3. Vaccination Readiness	4.36	0.88	.92	−.34**	.85**										
4. Behavior Change Intention	4.18	0.73	.86	−.14*	.44**	.48**									
5. Attitudes Towards Public Measures	3.72	1.18	.94	−.11	.45**	.51**	.59**								
6. Threat Belief	4.96	0.87	.88	−.28**	.56**	.63**	.59**	.56**							
7. Efficacy Belief (general)	4.65	0.96	.80	−.29**	.60**	.62**	.57**	.49**	.68**						
*a. Individual Efficacy Belief (general)*	4.49	1.05	.75	−.24**	.53**	.55**	.52**	.47**	.59**	.93**					
*b. Collective Efficacy Belief (general)*	4.90	1.09	.64	−.30**	.55**	.57**	.50**	.39**	.66**	.85**	.60**				
8. Efficacy Belief (vaccination)	4.88	1.11	.89	−.33**	.81**	.82**	.46**	.48**	.60**	.76**	.71**	.64**			
*a. Individual Efficacy Belief (vaccination)*	4.76	1.23	.89	−.32**	.78**	.78**	.45**	.45**	.56**	.75**	.74**	.59**	.96**		
*b. Collective Efficacy Belief (vaccination)*	5.07	1.15	.69	−.30**	.73**	.73**	.41**	.44**	.56**	.63**	.54**	.61**	.89**	.72**	
9. Political ideology	2.44	1.10	–	.39**	−.43**	−.51**	−.24**	−.28**	−.40**	−.39**	−.31**	−.41**	−.42**	−.38**	−.42**

*N* = 271. Pearson correlations were conducted. Entity World Belief refers to participants’ responses on the manipulation check. Higher values of political ideology represent a stronger conservative (vs. liberal) view. * *p* < .05, ** *p* < .01.

**Novel pandemic scenario.** The description of the novel pandemic was divided into two parts: the first part introduced the so-called *SciX virus*, its way of transmission, and the type and severity of its symptoms. The second part of the scenario discussed the so-called *Scimed* vaccine, its effectiveness against catching and transmitting the SciX virus, and its potential side effects (the full scenario text can be found in the supplement).

The SciX virus resembled Covid-19 in that it spread to new countries quickly and was contagious for up to 20 days, even with no symptoms. The virus was similarly more serious for high-risk groups and less serious for the low-risk groups (which participants were told they belonged to). Furthermore, similar preventive measures were introduced by the government (e.g., social distancing). We described that the Scimed vaccine was introduced shortly after the SciX virus was discovered and had a similar efficacy level as the Pfizer-Biontech vaccine [34].

The SciX virus differed from Covid-19 in that it transmitted through physical contact (which required people to wear gloves). Furthermore, symptoms were different. In the milder course of the disease, people experience headaches, coughs, and skin rashes for a few days. The severe course of the disease affects the veins, potentially blocking blood supply to the heart and causing heart attacks.

**Vaccine readiness.** We measured vaccination readiness (i.e., whether people were ready to get vaccinated) with the Vaccine Readiness Scale [35]. It consists of 7 subscales (confidence, complacency, constraints, calculation, collective responsibility, compliance, and conspiracy) that are each measured by 3 items (such as “I sometimes miss out on vaccination because vaccination is bothersome” and “I also get vaccinated because I am thereby protecting other people). Participants were asked to think about the Scimed vaccine when responding to the items on a 6-point Likert scale from *Strongly disagree* [1] to *Strongly agree* [6]. We created an aggregated score of the scale in a way that higher scores reflect higher vaccine readiness.

**Efficacy beliefs about vaccination.** We used the same 5 items that were used to measure efficacy beliefs about general measures regarding the scenario but adapted them to focus on the perceived efficacy of vaccination (e.g., “I feel that if I get vaccinated against the SciX virus, it is only a ‘drop in the bucket’ and won’t make a difference.”). Participants responded on a 6-point Likert scale from *Strongly disagree* [1] to *Strongly agree* [6]. Like the scale on efficacy beliefs about general mitigation efforts, 3 items referred to people’s efficacy beliefs about individual efforts (e.g., “I feel that through getting vaccinated I can…”), and 2 items referred to efficacy beliefs about collective efforts (e.g., “If humans get vaccinated…”). We created aggregated scores for the scale (and the individual and collective efficacy subscales) in a way that higher scores represent higher efficacy beliefs.

### Results

#### Preliminary results.

The manipulation check revealed a significant difference in reported lay beliefs about the world between the two experimental groups. As expected, the incremental group reported lower agreement (*M* = 3.3, *SD* = 1.2) with an entity belief about the world compared to the entity group (*M* = 3.7, *SD* = 1.3), *t*(263.65) = −2.53, *p* = .006, *d* = 0.31. Besides, the groups did not differ in gender, ethnicity, or political orientation (*p* > .645). However, they did differ in age (*M*_*incremental*_ = 35.5, *SD* = 12.7, *M*_*entity*_ = 38.9, *SD* = 14.7), *t*(256) = −2.03, *p* = .044. The resul*t*s did not change substantially when controlling for age (see supplement for the full results). Thus, we report the analyses without controlling for age.

All pandemic-related belief, attitude, and behavior variables were significantly correlated among each other (Table 5). Besides, lay beliefs about the world related to almost all pandemic-related variables: a stronger entity belief about the world correlated with lower vaccination intention and readiness, less intended behavior change and lower threat and efficacy beliefs (Table 5).

#### Pandemic-related outcomes.

There were no significant differences between the incremental and entity group regarding any of the pre-registered behavioral inclinations, beliefs, or attitudes regarding the pandemic scenario (see Table 6).

A further exploratory analysis revealed significant differences specifically for the two efficacy subscales that focused on individual behavior. People in the incremental group reported higher efficacy beliefs about individual mitigation efforts (*M* = 4.6) than people in the entity group (*M* = 4.4), *t*(262.45) = 1.79, *p* = .037, *d* = −0.22. Likewise, the incremental group reported higher efficacy beliefs about individual vaccination (*M* = 4.9) compared to the entity group (*M *= 4.6), *t*(252.83) = 1.79, *p* = .037, *d* = −0.22.

**Table 6:** Group Differences Between the Incremental and Entity Group Regarding the Pandemic-Related Outcomes in Study 3a.

Outcomes	*M*_incremental_	*M*_entity_	*t*	*df*	*p*	*d*
Vaccination intention^1^	4.4	4.3	0.92	255.29	.179	−0.11
Vaccine readiness^1^	4.4	4.3	0.65	257.96	.257	−0.08
Behavior change intention^1^	4.1	4.2	−1.15	268.25	.875	0.14
Threat belief^1^	5.0	5.0	0.08	256.69	.468	−0.01
Efficacy beliefs about mitigation efforts^1^	4.7	4.6	0.84	260.97	.199	−0.10
Efficacy beliefs about *individual* mitigation efforts	4.6	4.4	1.79	262.45	.037*	−0.22
Efficacy beliefs about vaccination^1^	5.0	4.8	1.21	256.39	.113	−0.15
Efficacy beliefs about *individual* vaccination	4.9	4.6	1.79	252.83	.037*	−0.22
Attitudes towards public measures^1^	3.7	3.7	0.04	264.48	.484	−0.01

*N *= 271. One-sided t-tests were conducted testing for higher values in the incremental belief group. ^1^Pre-registered analyses. * *p* < .05.

### Discussion

The aim of Study 3a was to test if participants that were led to adopt an incremental belief about the world (rather than an entity belief about the world) would report [1] stronger perceived seriousness of a novel pandemic, [2] stronger belief in the efficacy of mitigation efforts, [3] more supportive attitudes towards governmental measures against the pandemic, and [4] stronger behavioral intention concerning mitigation behavior. Study 3a did not find support for this. However, we found that participants in the incremental belief condition reported stronger efficacy beliefs about *individual* efforts specifically. There was also a trend indicating differences in intention to get vaccinated. A possible reason why we did not find the hypothesized effects might be that the manipulation was not strong enough. This is corroborated by the small effect on the manipulation check itself. The effects on vaccine intention and the full efficacy scales were smaller than those assumed in our power analysis, potentially leading to an insufficient sample size to detect these effects. Therefore, strengthening the manipulation might be necessary. The outcome variables were also highly skewed, and it can be difficult to find an effect if there is little variance in the responses. We address these points in Studies 3b-c.

## Study 3b

Study 3b aimed to replicate the effect of lay beliefs about the world on efficacy beliefs about individual mitigation efforts and vaccination. It also aimed to show an effect on vaccine intention by strengthening the manipulation of lay beliefs about the world. The manipulation was reinforced with a saying-is-believing exercise, which is a method to increase the internalization of previously received messages [36].

### Method

#### Participants.

The study was pre-registered here: https://aspredicted.org/2QL_QFG. A power analysis determined a sample of 278 participants to obtain a power of 80% with a standard significance level of α = 0.05, to detect an effect of *d* = 0.3 on a one-sided *t*-test. We recruited 304 people from the US via Prolific (recruiting period: 11/04/2022–19/04/2022) to account for exclusion of those who failed an attention check (*N* = 7), a reading comprehension question (*N* = 4) or indicated that their data should not be used (self-exclusion; *N* = 4). We analyzed 290 participants’ data (49% female, 49% male, 1% non-binary; mean age 41 years, *SD* = 13.9). Participants were mostly White (78%) with others being Asian (8%), Hispanic (8%), Black (4%) or another ethnicity (2%). The sample slightly tended to be politically liberal (*M* = 2.4, *SD* = 1.2 on a scale from *Very liberal* [1] to *Very conservative* [5]). The sample was evenly distributed across the randomly assigned experimental groups (*N*_incremental_ = 147, *N*_entity_ = 143).

#### Procedure and measures.

The procedure was the same as in Study 3a. An additional exploratory block was added at the end of the study. The full material including the exploratory block can be found under the following link: https://osf.io/57rpw/?view_only=2ef554e052b94b4b9335d1cdf1eafbcb. Descriptive statistics of the main variables are displayed in Table 7.

**Table 7:** Correlation Matrix of the Main Variables in Study 3b.

Variable	*M*	*SD*	α	1	2	3	4	4a	4b	5	5a	5b
1. Entity World Belief	3.41	1.29	.92									
2. Vaccination Intention	4.10	1.27	–	−.25**								
3. Vaccination Readiness	4.29	0.95	.93	−.33**	.87**							
4. Efficacy Belief (general)	4.48	1.13	.91	−.40**	.53**	.59**						
*a. Individual Efficacy Belief (general)*	4.22	1.19	.82	−.35**	.47**	.53**	.95**					
*b. Collective Efficacy Belief (general)*	4.74	1.18	.88	−.42**	.54**	.59**	.95**	.80**				
5. Efficacy Belief (vaccination)	4.78	1.20	.95	−.40**	.77**	.80**	.80**	.72**	.79**			
*a. Individual Efficacy Belief (vaccination)*	4.58	1.27	.91	−.37**	.71**	.75**	.78**	.75**	.73**	.97**		
*b. Collective Efficacy Belief (vaccination)*	4.98	1.22	.91	−.40**	.77**	.80**	.76**	.65**	.80**	.96**	.86**	
6. Political ideology	2.40	1.16	–	.30**	−.43**	−.52**	−.44**	−.35**	−.49**	−.49**	−.42**	−.52**

*N* = 290. Pearson correlations were conducted. Entity World Belief refers to participants’ responses on the manipulation check. Higher values of political ideology represent a stronger conservative (vs. liberal) view. * *p* < .05, ** *p* < .01.

**Experimental manipulation.** The first part of the experimental manipulation was the same as in Study 3a: participants read a passage about whether the world was rather dynamic or fixed. After that, participants were asked to come up with reasons why the world is changing or fixed (depending on the experimental condition) and why this relates to their life (saying-is-believing exercise). As in the previous studies, we used the lay beliefs about the world scale as a manipulation check.

**Behavior intention.** Vaccination intention and vaccine readiness were both measured with the same scales as in Study 3a.

**Efficacy beliefs about general mitigation efforts.** We used the same efficacy belief scale as in Study 3a but added another item to the efficacy of collective action subscale, to even out the number of items of the two subscales. Furthermore, we slightly changed the wording of the items to make them stronger in order to reduce the negative skewness found in the previous study. For example, “I feel that through my personal behavior, I can influence the course of the SciX virus pandemic” was changed to “I feel that through my personal behavior, I can *highly* influence the course of the SciX virus pandemic”. We created aggregated scores for the separate subscales (regarding individual vs. collective mitigation efforts) in a way that higher scores represent higher efficacy beliefs.

**Efficacy beliefs about vaccination.** The scale was the same as we used in Study 3a, but we added another item to the efficacy of collective vaccination efforts subscale, to even out the number of items on the two subscales. We created aggregated scores for the separate subscales (regarding individual vs. collective vaccination efforts) in a way that higher scores represent higher efficacy beliefs.

### Results

#### Preliminary results.

The manipulation check revealed a significant difference in reported lay beliefs about the world between the two experimental groups. The incremental belief group reported lower agreement (*M* = 3.0, *SD* = 1.1) with an entity belief about the world compared to the entity belief group (*M* = 3.8, *SD* = 1.3), *t*(280.18) = −5.99, *p* < .001, *d* = 0.71. The effect size was medium to large, indicating that we successfully strengthened the manipulation compared to Study 3a (*d* = 0.31). Besides, the groups did not differ in gender, age, or political orientation (*p* > .430). However, they differed in ethnicity (Fisher’s exact test, *p* = .035). The results did not change, when controlling for ethnicity. Thus, we report the results of the analyses without controlling for ethnicity.

All pandemic-related belief, attitude, and behavior variables were significantly correlated among each other (Table 7). Again, lay beliefs about the world related to all pandemic-related variables: a stronger entity belief about the world correlated with lower vaccination intention and readiness, and lower efficacy beliefs (Table 7).

#### Pandemic-related outcomes.

There were no significant differences between the incremental and entity belief groups regarding any of the pre-registered outcomes (see Table 8). Specifically, the two groups did not differ in their vaccination intention or in their efficacy belief with regard to individual mitigation efforts or individual vaccination. There was neither a group difference on any of the other pandemic-related outcomes that were collected for exploratory purposes (see Table 8).

**Table 8:** Group Differences Between the Incremental and Entity Group Regarding the Pandemic-Related Outcomes in Study 3b.

Outcomes	*M*_incr_	*M*_ent_	*t*	*df*	*p*	*d*
Vaccination intention^1^	4.1	4.1	0.17	287.81	.434	−0.02
Efficacy beliefs about general *individual* mitigation efforts^1^	4.3	4.2	0.65	287.91	.259	−0.08
Efficacy beliefs about *individual* vaccination^1^	4.6	4.6	−0.05	282.06	.518	0.01
Vaccine readiness	4.3	4.3	0.47	286.97	.320	−0.06
Efficacy beliefs about general *collective* mitigation efforts	4.8	4.7	1.19	287.20	.118	−0.14
Efficacy beliefs about *collective* vaccination efforts	5.0	5.0	0.02	285.96	.492	0.00

*N* = 290. One-sided t-tests were conducted testing for higher values in the incremental group. ^1^Pre-registered analyses.

### Discussion

As was the goal in Study 3b, the manipulation got stronger by integrating a saying-is-believing task into the protocol and the distribution of the outcome measures were less skewed. However, we were not able to replicate our findings from Study 3a. This may have several reasons. One could be that we dropped the measures on perceived threat and behavior change intention from Study 3a. These additional measures contained some nuanced information about the pandemic scenario. For instance, the intended behavior change measure included information about what type of behaviors can be effective in this situation, which might have led people to think more deeply about the possible benefits of their behaviors (i.e., the efficacy of their mitigation efforts). Therefore, those measures will be reintroduced in Study 3c while also collecting a larger sample.

## Study 3c

The aim of Study 3c was to directly replicate Study 3a on a bigger sample, while keeping all measures that could add nuanced information about the scenario. As we only found a significant effect on efficacy beliefs about mitigation efforts and getting vaccinated (and not vaccine intention), our pre-registered hypothesis referred to only these two variables (and not vaccine intention). We expected that those participants who would be exposed to the incremental belief about the world manipulation, would have stronger efficacy beliefs about mitigation efforts and about getting vaccinated.

### Method

#### Participants.

The study was pre-registered under the following link: https://aspredicted.org/HKF_RCD. A power analysis determined a sample of 514 participants to obtain a power of 80% with a standard significance level of α = 0.05, to detect an effect of *d* = 0.22 on a one-sided *t*-test (based on the effects found in Study 3a). The study was conducted 24/05/2022–26/05/2022 in a US sample via MTurk. We obtained 627 unique complete responses after excluding responses from 21 people who participated more than once (each time the first response was retained, and later responses were removed). We excluded those who failed an attention check (*N* = 94), a reading comprehension question (*N* = 0) or indicated that their data should not be used (self-exclusion; *N* = 9). Finally, we analyzed 524 participants’ data (69% female, 28% male, 2% non-binary; mean age = 38 years, *SD* = 12.3). Participants were mostly White (73%) with others being Black (12%), Asian (7%), Hispanic (5%), Native American (1%) or another ethnicity (2%). The sample slightly tended towards being politically liberal (*M* = 2.7, *SD* = 1.1 on a scale from *Very liberal* [1] to *Very conservative* [5]). The sample was evenly distributed across the randomly assigned experimental groups (*N*_incremental_ = 264, *N*_entity_ = 260).

#### Procedure and measures.

The procedure was the same as in Study 3a. The full material of the study can be found under the following link: https://osf.io/57rpw/?view_only=2ef554e052b94b4b9335d1cdf1eafbcb. Descriptive statistics of the main variables can be found in Table 9. The measures were the same as in Study 3a, but the threat belief scale was reduced to 4 items to decrease the length of the study.

**Table 9:** Correlation Matrix of the Main Variables in Study 3c.

Variable	*M*	*SD*	α	1	2	3	4	5	6	7	7a	7b	8	8a	8b	9
1. Entity World Belief	3.51	1.25	.91													
2. Vaccination Intention	3.80	1.45	–	−.19**												
3. Vaccination Readiness	3.99	1.05	.93	−.26**	.87**											
4. Behavior Change Intention	4.03	0.88	.90	−.19**	.41**	.52**										
5. Attitudes Towards Public Measures	3.99	1.34	.91	−.16**	.48**	.59**	.73**									
6. Threat Belief	4.75	0.93	.81	−.21**	.41**	.54**	.52**	.53**								
7. Efficacy Belief (general)	4.57	1.06	.89	−.32**	.50**	.60**	.57**	.55**	.56**							
*a. Individual Efficacy Belief (general)*	4.40	1.13	.81	−.32**	.47**	.55**	.56**	.53**	.51**	.93**						
*b. Collective Efficacy Belief (general)*	4.73	1.14	.83	−.28**	.47**	.58**	.51**	.49**	.54**	.94**	.75**					
8. Efficacy Belief (vaccination)	4.62	1.26	.93	−.29**	.80**	.85**	.49**	.51**	.53**	.73**	.68**	.68**				
*a. Individual Efficacy Belief (vaccination)*	4.49	1.37	.91	−.29**	.79**	.83**	.47**	.50**	.49**	.70**	.69**	.63**	.96**			
*b. Collective Efficacy Belief (vaccination)*	4.76	1.26	.86	−.27**	.74**	.80**	.47**	.48**	.52**	.69**	.61**	.69**	.95**	.83**		
9. Political ideology	2.68	1.14	–	.17**	−.43**	−.53**	−.32**	−.38**	−.36**	−.39**	−.32**	−.40**	−.45**	−.42**	−.45**	
10. Trust	3.04	0.87	.77	−.15**	.54**	.60**	.28**	.29**	.30**	.37**	.34**	.35**	.56**	.54**	.53**	−.27**

*N* = 524. Pearson correlations were conducted. Entity World Belief refers to participants’ responses on the manipulation check. Higher values of political ideology represent a stronger conservative (vs. liberal) view. * *p* < .05, ** *p* < .01.

**Influence of Covid-19 views.** Two questions assessed to what degree participants’ responses were influenced by their views about Covid-19. First, participants indicated what rather influenced their answers to the questions on the SciX virus on a 5-point scale (*The information you read about the SciX virus* [1]; *Both equally* [3]; *Your views about Covid-19* [5]). Second, they indicated how similar their answers had been to what they would have answered to questions about Covid-19 on a 5-point scale (*Very different answers* [1] to *Exactly the same answers* [5]).

**Trust.** Participants indicated their trust in the US government, their state’s government, and scientists in general on a scale from *Cannot be trusted at all* [1] to *Can be trusted a lot* [5]. We averaged these items in a way that higher scores represent higher trust (α = .77, *M* = 3.04, *SD* = 0.87).

#### Statistical analysis.

Differences between the two experimental groups (on both pre-registered and exploratory outcomes) were tested with one-sided *t*-tests as in the previous studies.

Beyond that, we conducted an exploratory network analysis to explore the correlational relationship of measured lay beliefs of the world (as opposed to the effect of the manipulation). The network analysis was performed with R 4.3.2 [33]. We residualized the group’s effect on all pandemic-related variables to partial out any effect the manipulation had on the variables of interest. As efficacy beliefs about general mitigation efforts and efficacy beliefs about vaccination highly correlated (*r*_*Pearson*_ = .73, *p* < .001), we collapsed these two scales for the network analysis. The network analysis aimed to explore how lay beliefs about the world are related to other constructs in the network structure of the pandemic-related behavioral intentions, attitudes, and beliefs. We used the EBICglasso method to generate the network, with a tuning parameter of 0.5 which is a conservative method that helps to avoid spurious edges [37,38]. In line with recommendations [39], a rank-transformation was conducted before estimating the network because not all data was normally distributed. According to literature [37], the most widely used psychological network analysis technique is the partial correlation network (which can be conducted using the EBICglasso methodology). Partial correlation networks estimate partial correlation coefficients ranging from −1–1 representing the remaining association between two constructs after controlling for all other variables in the model [37].

### Results

#### Preliminary results.

The manipulation check revealed a significant difference in lay beliefs about the world between the two experimental groups. The incremental group reported lower agreement (*M* = 3.3, *SD* = 1.2) with an entity belief about the world compared to the entity group (*M* = 3.7, *SD* = 1.3), *t*(516.40) = −4.11, *p* < .001, *d* = 0.36. Besides, the groups did not differ in gender, ethnicity, age, or political orientation (*p* > .347).

All pandemic-related belief, attitude and behavior variables were significantly correlated among each other (Table 9). Besides, as in the previous studies lay beliefs about the world related to all pandemic-related variables: a stronger entity belief about the world correlated with lower vaccination intention and readiness, less intended behavior change, more negative attitudes towards public measures and lower threat and efficacy beliefs (Table 9).

Most participants indicated that they based their answers to some degree on their pre-existing views about Covid-19. The majority (45%) reported their answers to be equally influenced by the presented information and Covid-19. Further 16% reported to be rather or even only influenced by Covid-19. Additionally, most people (81%) reported that they gave mostly or exactly the same answers that they would give to questions about Covid-19. Thus, the scenario did not create a situation where participants would newly form beliefs, attitudes, and intentions as we had hoped.

#### Pandemic-related outcomes.

There were no significant differences between the incremental and entity belief groups regarding the two pre-registered outcomes: efficacy beliefs about general mitigation efforts and efficacy beliefs about vaccination (see Table 10). There was neither a group difference on any of the other pandemic-related outcomes that were collected for exploratory purposes (see Table 10).

**Table 10:** Group Differences Between the Incremental and Entity Belief Groups Regarding the Pandemic-Related Outcomes in Study 3c.

Outcomes	*M*_incremental_	*M*_entity_	*t*	*df*	*p*	*d*
Efficacy beliefs about mitigation efforts^1^	4.5	4.6	−0.43	520.83	.666	0.04
Efficacy beliefs about getting vaccinated^1^	4.6	4.6	−0.09	521.95	.534	0.01
Vaccination intention	3.8	3.8	−0.07	521.77	.527	0.01
Vaccine readiness	4.0	4.0	−0.28	521.83	.611	0.02
Behavior change intention	4.0	4.1	−0.81	520.47	.790	0.07
Threat belief	4.7	4.8	−0.18	521.99	.570	0.02
Attitude towards measures	4.0	4.0	−0.43	519.89	.667	0.04

*N* = 524. One-sided *t*-tests were conducted, testing for higher values in the incremental group. ^1^Pre-registered analyses.

#### Network analysis.

As all previous studies showed correlational relationships between measured lay beliefs about the world and the pandemic-related outcomes, we conducted a network analysis on these variables to learn more about these relationships. We included measured lay beliefs about the world, general measures (age, political orientation, and trust) and all pandemic-related variables (vaccination intention, the subscales of vaccination readiness, intended behavior change, threat and efficacy beliefs, and attitudes).

**Fig 1:**
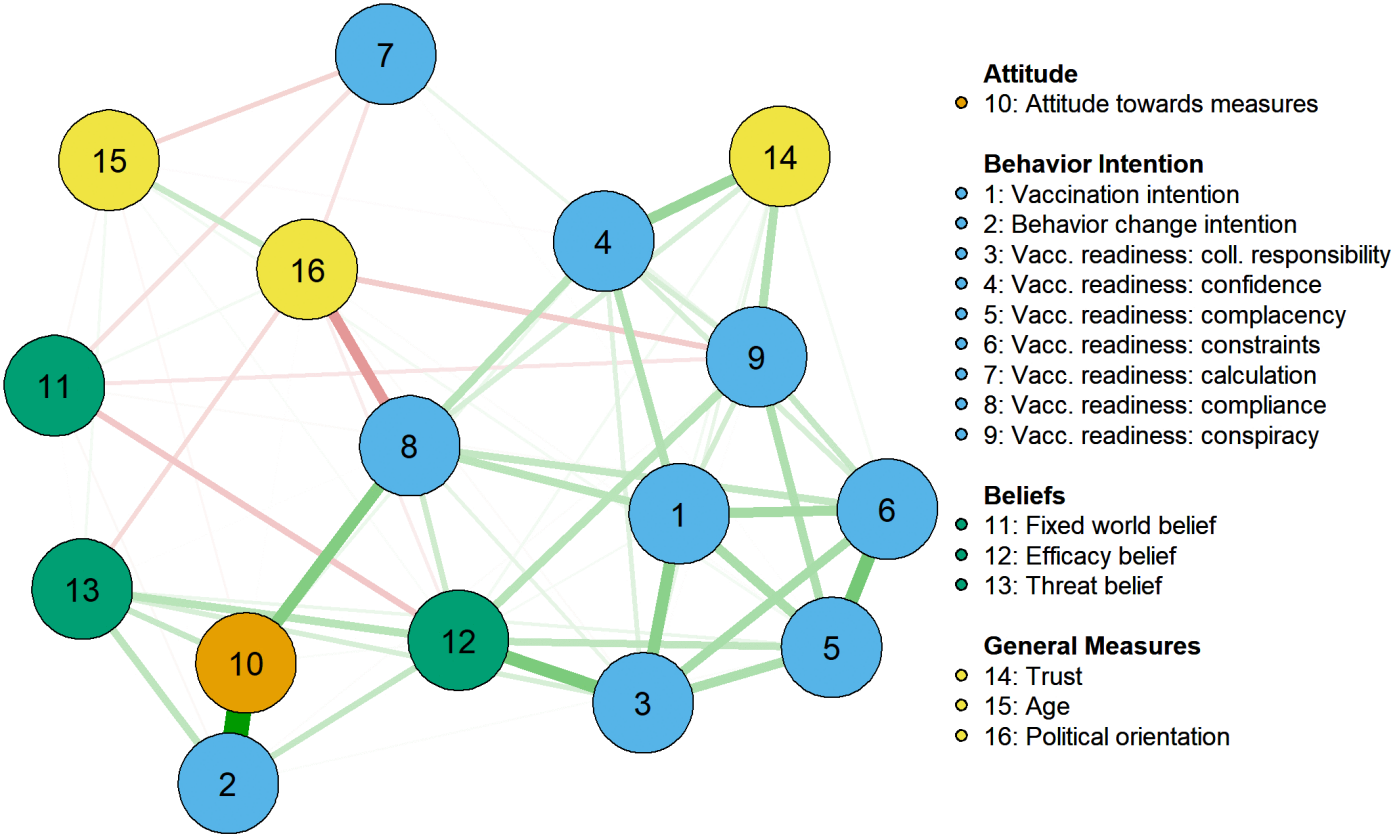
Plot of the Network Analysis on Pandemic-Related Behaviors, Attitudes, Beliefs and General Measures.

**Fig 2:**
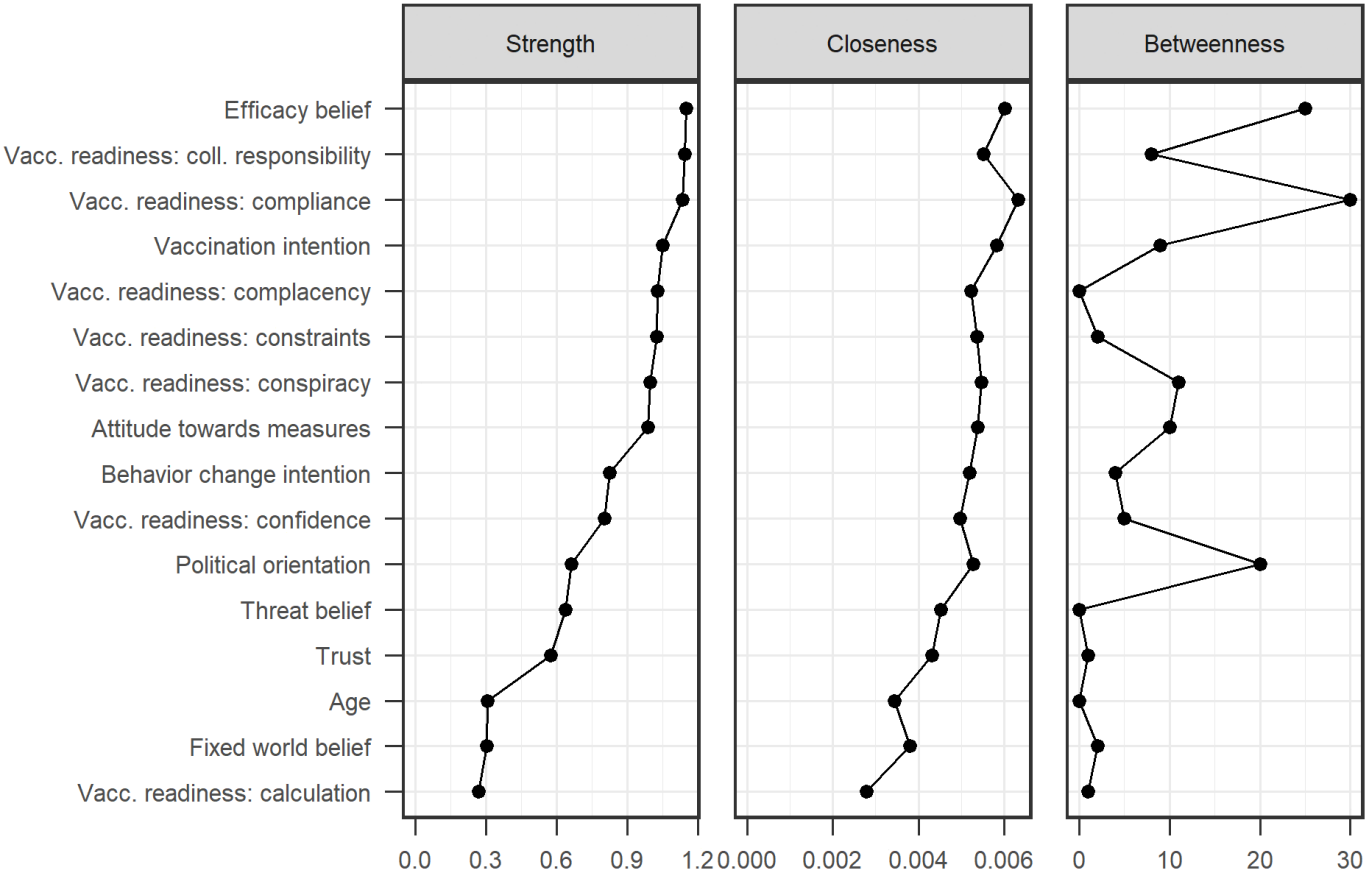
Centrality Plot of the Network Analysis on Pandemic-Related Behaviors, Attitudes, Beliefs, and General Measures.

The resulting network structure is shown in Fig 1 color-coded by type of measure (attitude, behavior intention, beliefs, and general measures). Fig 2 displays centrality measures of the nodes in the network (higher numbers reflecting more central nodes). The network structure shows that the items related to vaccination appear in a strong hub, which is connected to efficacy beliefs, attitudes, trust, and political orientation. Interestingly, threat belief and behavior change intention have no strong, direct associations to vaccination-related items, but indirect ones through efficacy beliefs and attitudes. Lay beliefs about the world and age are only connected to few of the other variables.

The centrality plots show that efficacy beliefs are very central to the network, being connected to most outcomes (i.e., behavior intentions and threat belief). Threat belief is only related to general behavior change intention and attitudes–however, it is not related to vaccine-related behaviors. Lay beliefs about the world show one of the lowest centralities in the network, having its strongest connection to efficacy beliefs. Among the non-pandemic-related variables, trust and political ideology have the highest centralities, both being mainly related to vaccination-related items.

### Discussion

Our effort to replicate Study 3a on a bigger sample was unsuccessful. We expected that those participants who would be exposed to the incremental belief about the world manipulation, would have stronger beliefs in the efficacy of mitigation efforts and the efficacy of getting vaccinated. However, we found no such effects. Yet, our exploratory network analysis revealed interesting, correlational results that we would like to highlight three points from.

First, lay beliefs about the world add unique variance but are not central to the network. They connect directly to efficacy beliefs, and efficacy beliefs then mediate their relationship to most other variables (e.g., behavior intentions). Since efficacy beliefs are a central node in the network and related to several of the pandemic-related outcomes, the relationship between lay beliefs about the world and efficacy beliefs might still be interesting to investigate. It might provide insights about how efficacy beliefs develop.

Second, according to the Health Belief Model, perceived benefits of one’s behavior and perceived seriousness of a health threat are important determinants of health behavior. Perceived benefits as measured by the efficacy belief scales in our study in fact appear to be very central in the network. They have the highest strength of direct connections and are connected to important outcomes such as vaccination intention and readiness as well as general behavior change intention (however not to attitudes about public measures). Perceived seriousness, as measured by the threat belief scale, was less central to the network. Still, it was directly related to general behavior change intention and attitudes about public measures, but less so to vaccination intention and readiness.

The third key finding of the network analysis is the role of vaccination intention, which has been the most important dependent variable to our paper. Aside from the variables directly related to vaccine readiness, only efficacy beliefs and trust have a direct connection to vaccination intention. On the one hand, this shows that increasing efficacy beliefs might be important to achieve behavioral changes (e.g., getting vaccinated) in a pandemic. If people feel that their behavior can influence the course of the pandemic, they also tend to act towards this goal. On the other hand, trust play an important role in vaccination (also shown by its connections to vaccine readiness). It is crucial to trust officials because people might not have the knowledge to fully understand information about vaccines, however if they trust important authorities (scientists or the government), they are also more likely to get vaccinated if it is recommended in a pandemic scenario.

The correlational analyses of the previous studies show that people’s lay beliefs about the world are connected to pandemic-related behaviors, behavior intentions, attitudes, and beliefs. The network analysis in study 3c further shows that people’s lay beliefs about the world are still associated with pandemic-related efficacy beliefs (and through that with other outcomes) if general measures (e.g., age, political orientation, and trust) and other pandemic-related variables are held constant. However, there were no reliable effects of manipulated lay beliefs about the world on the same outcomes. This opens the question about the underlying causality of the relationship. Possibly, lay beliefs about the world are in fact not a driver of pandemic-related outcomes, but instead lay beliefs about the world are influenced by people’s existing views about Covid-19 or pandemics in general. This will be tested in Study 4.

## Study 4

The aim of Study 4 was to investigate a possible reversed causal effect, indicating that people might shift their lay beliefs about the world to justify a certain behavior. All previously reported studies find correlations between measured lay beliefs about the world and pandemic-related outcomes, such as behavior and behavior intentions. However, we found no evidence of a causal effect of lay beliefs about the world on these outcomes. A possible explanation is that lay beliefs about the world do not shape people’s behavior, but instead behavior shapes people’s lay beliefs because people adopt a certain lay belief to justify their behavior or inaction. Past research shows that people sometimes shift their lay beliefs to support a personally preferred conclusion (e.g., justify a behavior or support a certain reasoning) [40]. For example, people shifted towards a more incremental belief about personal characteristics after receiving failure feedback or recalling a personal social failure, which would support their personally preferred conclusion (i.e., “I can get better” instead of “I will stay bad at this”). Similarly, people who made a big effort to adhere to the guidelines might be motivated to reassure themselves that this was not a waste by adopting an incremental belief. In contrast, people who did not adhere thoroughly to Covid-19 related guidelines might justify this by adopting an entity belief about the world to avoid appearing reckless. In Study 4, we test this process of motivated reasoning by asking participants to imagine a certain behavior (either reflecting high or low adherence with protective guidelines) and measuring the effect this has on their lay beliefs about the world.

### Method

#### Participants.

The study was pre-registered under https://aspredicted.org/9B4_X2B. A power analysis determined a sample of 398 participants to obtain a power of 80% with a standard significance level of α = 0.05, to detect an effect of *d* = 0.25. To account for exclusions, we aimed for a sample size of *N* = 420. Overall, 421 participants from the US were recruited through Prolific (on 11/08/2023). Participants’ data was excluded if they indicated that their data should not be used (*N* = 1), failed a reading check (*N *= 2) or failed an attention check (*N* = 8). Thus, the final sample size was *N* = 410. The sample consisted equally of men (50%) and women (49%; 1% non-binary). Most participants were White (66%) followed by Black (20%; 5% were Asian and Hispanic each, 1% was Native American and 4% indicated another race). Participants were on average 45 years old (*SD* = 13.6) and rather politically liberal (*M* = 2.6, *SD* = 1.3 on a scale from *Very liberal* [1] to *Very conservative* [5]). The sample was evenly distributed across the randomly assigned experimental groups (*N*_*low adherence*_ = 207, *N*_*high adherence*_ = 203).

#### Procedure and measures.

After participants gave written consent to the participation information and the data protection form, they read the first part of the same pandemic scenario that was used in Studies 3a-c. This part introduced the so-called *SciX virus*, its way of transmission, and the type and severity of its symptoms. Afterwards, participants answered two reading check items. Next, the manipulation was administered: participants read a scenario that asked them to imagine either enacting or failing to enact protective health behavior. Following this, participants answered questions regarding the scenario, including a manipulation check. Then, participants completed a questionnaire on different kinds of lay beliefs, including lay beliefs about the world. Questions on other lay beliefs were included to mask the focus of the study. Finally, participants answered control questions on how well they were able to imagine the provided scenario and were thanked and debriefed. The full material can be found under https://osf.io/57rpw/?view_only=2ef554e052b94b4b9335d1cdf1eafbcb.

**Manipulation: adherence scenario.** To manipulate participants’ motive to shift their lay beliefs about the world either towards an entity or incremental belief, we asked them to imagine a concrete situation set within the described pandemic scenario. The first part of the description was identical for both groups: “*You have an important event coming up. Due to the number of people attending (including vulnerable individuals), you plan to test for the SciX virus before the event to ensure everybody’s safety, although it isn’t mandatory.*” The second part differed between the groups. The high adherence scenario group read: “*On the day of the event, you get tested in the morning. The tests typically take 3-4 hours to be analyzed. However, this time it seems to take longer, and you fear you might not get the results in time. Fortunately, you end up receiving the result one hour before the event. As the result is negative, you go to join the event*.” Instead, the low adherence scenario group read: “*On the day of the event, just an hour before the start, you suddenly realize that you forgot to take the test. You know there is no time to get tested and receive the results in time for the event. The tests typically take 3-4 hours to be analyzed. You debate what to do and finally decide to go to the event without being tested*.”

**Manipulation check.** To ensure that the manipulation successfully gave participants a motive to justify their lack of protective behavior, we asked participants how they would feel in the described situation. Participants completed the Positive and Negative Affect Scale (PANAS) [41]. They reported to what extent they would feel certain emotions (e.g., ashamed, enthusiastic) on a scale from *very slightly or not at all* [1] to *extremely* [5]. Separate scores for positive (α = .92) and negative (α = .92) affect were calculated.

**Lay beliefs about the world.** The same measure as in Study 1 was used (α = .94).

**Control questions.** After the manipulation check, participants were asked to indicate to what extent they perceived the outcome of the situation to be influenced by themselves (*0% influenced by you* (0) to *100% influenced by you* (100)). This was done to ensure that people felt responsible for the described behavior. At the end of the study, participants were asked how vividly they could imagine the described situation, how immersed they felt in it, and how likely something similar would happen to them (from *not at all vividly/ immersed/ very unlikely* [1] to *very vividly/ immersed/ likely* [5]).

#### Statistical analysis.

In line with our pre-registration, we conducted a one-sided *t*-test to test whether the low adherence group reported more negative affect as a manipulation check. Next, we conducted a second one-sided *t*-test to test if the low adherence group reported a stronger entity belief about the world compared to the high adherence group.

### Results

#### Preliminary results.

The manipulation check showed that the manipulation was successful. Participants in the low adherence group reported that they would experience stronger negative affect in the situation (*M* = 2.8, *SD* = 0.94) than the high adherence group (*M *= 1.8, *SD* = 0.71), *t*(383.4) = 11.9, *p* < .001, *d* = −1.2. Additionally, participants in the low adherence group reported lower positive affect (*M* = 2.4, *SD* = 0.64) than the high adherence group (*M* = 2.5, *SD* = 0.70), *t*(403.0) = −2.8, *p *= .003, *d* = 0.28. Besides, *t*he experimental group did not differ in age, gender, ethnicity, or political ideology (*p* > .489).

Overall, participants felt that the outcome of the described scenario was by ca. 70% influenced by themselves (*M* = 68.9, *SD* = 28.8). On average, they could image the situation quite vividly (*M* = 4.2, *SD* = 0.84), felt quite immersed in it (*M* = 4.1, *SD* = 0.91) and considered it moderately likely that something similar would happen to them (*M* = 3.2, *SD* = 1.29).

#### Lay beliefs about the world.

Regarding the main hypotheses, we did not find the expected effect. Participants in the low adherence group did not show a significantly lower incremental belief about the world (*M* = 3.4, *SD* = 1.26) than the high adherence group (*M* = 3.5, *SD* = 1.36), *t*(404.4) = −0.98, *p* = .163, *d* = 0.10.

### Discussion

Study 4 found no evidence that people might shift their lay beliefs about the world to justify a certain behavior. Imaging a situation where one exhibits either high or low protective behavior did not lead to differences in participants’ lay beliefs about the world. However, this study does not provide enough evidence to make any final conclusions. Possibly, the manipulation was not strong enough to provide a motive to shift one’s belief. Participants in the low adherence group did report more negative affect (e.g., feeling guilty or ashamed), however, this referred to a situation that they knew was not real. An imagined behavior might not provide enough motivation to shift one’s lay belief. Actual behavior presents a much stronger need to be justified. Besides, the manipulation mainly provided a motive to adopt an entity belief (in case of low adherence). It did not provide a strong motive to adopt an incremental belief in case of high adherence because there were no costs associated with the high adherence. This might have limited the potential differences between the groups.

## General discussion

In the current paper, we provided initial evidence that people’s lay beliefs about the world might play a role in the fight against pandemic situations such as the global Covid-19 pandemic. However, the exact role it plays remains unclear. We did not find evidence for a causal effect of manipulated lay beliefs about the world on pandemic-related outcomes, nor a reversed causal effect (of pandemic-related behavior shaping one’s lay beliefs about the world). However, we consistently find correlational relationships between naturally occurring lay beliefs and pandemic-related outcomes.

In Study 1, a correlational analysis showed that lay beliefs about the world were related to several variables regarding behavior, beliefs, and attitudes concerning Covid-19 even when controlling for political ideology. Our aim in Study 2 was to show that inducing an incremental belief about the world would result in more favorable beliefs, attitudes, and behavior intentions regarding Covid-19. However, we did not find supporting evidence for this hypothesis. A possible explanation for this lack of evidence was that beliefs and attitudes around Covid-19 might have already been very fixed at the time of Study 2 due to a general polarization of the topic. Therefore, in Study 3a-c we created a hypothetical pandemic scenario to analyze the influence of manipulated lay beliefs about the world on people’s beliefs, attitudes, and behavioral intentions regarding a new health threat. In these studies, we found no evidence either that an incremental belief about the world would result in more favorable beliefs, attitudes, and behavior intentions. However, across all studies we found consistent correlations with measured lay beliefs of the world. Additionally, the network analysis in Study 3c supports that an incremental belief about the world is associated with more favorable behavioral outcomes indirectly through efficacy beliefs and to a lesser extent through threat belief.

Taken together, lay beliefs about the world were consistently associated with pandemic-related outcomes on the correlational level but did not show a causal effect. A possible explanation for this could be that in fact pandemic-related behavior influence people’s lay beliefs about the world and not the other way around. For example, people may try to justify their behavioral choices with the suitable lay belief about the world. Therefore, in Study 4 we tested a reversed causal effect of imagined behavior in a pandemic scenario on participants’ lay beliefs about the world. However, we did not find evidence for this effect either.

### Theoretical implications

Although there was no evidence of a causal effect of lay beliefs about the world, we consistently find that people who report a stronger incremental belief about the world also report more favorable beliefs, attitudes, and behavioral inclinations regarding both Covid-19 and the novel pandemic scenario. This indicates that there is some kind of relationship between them, at least on the correlational level. Past research has shown that an incremental belief about the world is positively related to acknowledging social issues (such as climate change), recognizing one’s personal impact on them, maintaining hope for the successful resolution, and addressing the issue appropriately [22–25]. Furthermore, past studies in the climate change domain [22,23] indicate that believing in climate change and believing in the efficacy of one’s mitigating efforts might act as mediators between lay beliefs about the world and behavioral intentions. With the current paper, we extend these results to the health domain. An incremental belief about the world related to acknowledging a global health threat (either Covid-19 or a novel, hypothetical pandemic) and recognizing one’s own impact on it. Thus, this study gives some indication that research on climate change can be helpful to understand other global scale problems, such as the Covid-19 pandemic. Besides that, this study extends past literature on the role of lay beliefs about the world to other social issues. Lay beliefs about the world seem to play a role in several large-scale, social problems where the personal relevance of the problem and one’s personal impact on it are ambiguous (be it climate change, political protest, or Covid-19). However, the nature of this relationship remains unclear. Our findings suggest that, at least in the context of a global health pandemic, there is not a simple causal relationship where changes in lay beliefs about the world translate directly to changes in the perceptions of a health threat.

The network analysis in Study 3c showed that lay beliefs about the world are rather peripheral in the overall system of pandemic-related outcomes (as indicated by their low centrality). Low centrality indicates that a variable is only connected to few other variables (e.g., lay beliefs about the world are mainly connected to efficacy beliefs). Low centrality also means that the variable can change rather independently of the other variables in the network [42]. Thus, small changes in lay beliefs about the world would not easily spread to other variables in the model. Instead, the different pandemic-related variables (i.e., beliefs, attitudes, and behavioral inclinations) were strongly interrelated among themselves (indicated by the medium to high centralities). This might lead to them being more difficult to change because they create a stable network that reinforces itself [42]. Essentially, the strong network of bidirectional relationships between the variables might suppress changes in each single variable–e.g., low efficacy belief might keep vaccination willingness low, but likewise low vaccination willingness might be rationalized through a low efficacy belief. This is corroborated by the fact that other general variables included in the model, such as political ideology or trust also seem to be less central in the model compared to the pandemic-related variables themselves. While non-central variables can still influence a network, they require larger changes to have an effect. Thus, it may need a substantial change in lay beliefs about the world for it to impact the other variables.

Lay beliefs about the world focus on the perception of the world as changeable. Past studies have also identified other ways in which people differ in how they perceive the world, e.g., perceiving it as just vs. unjust, harmless vs. dangerous, or beautiful vs. ugly [43]. These world beliefs have been shown to be considerably stable across a long period of time (e.g., 19 months) even with major events happening in the meantime (e.g., the 2016 US election). Possibly, several different world beliefs also form a somewhat robust worldview pattern that is learnt over time and sustains itself. This might make it difficult to change single parts of the worldview system.

Additionally, people’s views about Covid-19 are influenced by an interplay of views that are not Covid-19 specific, but, e.g., related to politics, science, or medicine in general. Our network analysis showed that political ideology and trust in the US government, the state’s government, and scientists were related to Covid-19 outcomes. Liberal participants and participants who reported higher trust displayed more favorable Covid-19 views. Other research shows that anti-establishment views and support for Donald Trump are also related to Covid-19 conspiracy beliefs beyond political ideology in terms of a liberal-conservative continuum [44]. Similarly, anti-vaccination views (e.g., regarding the measles, mumps, and rubella vaccination) existed before the surge of the Covid-19 pandemic [45,46] and Covid-19 attitudes might partially be a continuation of such previous views. Overall, people’s perceptions of Covid-19 might result not only from Covid-19 specific appraisals but also from preexisting views about politics or science in general.

#### Implications for the health belief model.

The results of this study do not only provide insights about the role of lay beliefs about the world, but also about the role of two important factors suggested by the Health Belief Model: perceived benefits of health behavior and perceived seriousness of the health threat. Our results highlight the relevance of perceived benefits in the form of efficacy beliefs. Efficacy beliefs were among the most central variables in the network model, while threat beliefs were less important. One explanation for this is that believing in the seriousness of the health threat might still not lead to health behavior if one does not believe that the behavior will effectively provide protection. Strong beliefs in the efficacy of a health behavior on the other hand might more consistently lead to engagement as long as the threat is perceived at least as somewhat serious – so to speak, “just to be safe”.

Our study also provides new insights that expand the Health Belief Model. Past studies have mostly focused on perceived benefits of a health behavior for the individual. However, in a global health threat such as the Covid-19 pandemic, benefits for others or the general society come into focus. Especially, when certain groups of people are less affected than others (such as young people being less vulnerable to Covid-19), these people might be motivated by benefits for others or the society rather than benefits for themselves. Our study focused on this aspect of perceived benefits (i.e., the efficacy of health behaviors in protecting others or alleviating the overall course of the pandemic) and showed that they were strongly related to people’s health behavior intentions. This indicates that people do not only take personal benefits into account but also benefits for others. This might prove useful to motivate people to engage in health behaviors that do not benefit them immediately. For instance, in a pandemic, it might be important to focus on the benefits of preventive behaviors and measures for the sake of helping other people and the society as a whole instead of just oneself.

While our research did not test the full Health Belief model, we used it as a guiding framework to motivate a focused examination of perceived severity and perceived benefits, at the collective level, under pandemic uncertainty. Overall, our results suggest that operationalizing these variables at the collective level adds new insight into the Health Belief literature. However, our research did not measure all components of the Health Belief Model. Further research could address this gap and explore whether other components of the Health Belief Model could add a new perspective at the collective level in health contexts at a global scale.

### Limitations and future research

Originally, we assumed that we did not find an effect of the manipulation on the Covid-19 related outcomes (Study 2) because lay beliefs about the world primarily exert their effect during the initial formation of beliefs, attitudes, and behavioral intentions but are less influential once these have formed and stabilized. However, the results from Studies 3a-c show that the manipulation neither affected people’s perceptions of a new health threat. Likewise, the strength of the manipulation did not seem to play a major role as the manipulation was successfully strengthened in study 3b without any changes in the effects on the outcomes.

A possible explanation is that the scenario we created was still too similar to the Covid-19 pandemic. Thus, people might have based their beliefs, attitudes, and behavioral inclinations regarding the new scenario on their pre-existing views regarding Covid-19. In that case, people did not thoroughly process the new information regarding the scenario but rather used their existing view of Covid-19 as a reference. We deliberately created a scenario that would be similar to the Covid-19 pandemic in order to be able to draw conclusions for the Covid-19 pandemic based on the scenario. However, a downside to this is that people did not perceive it as fundamentally new. In Study 3c, most people reported their answers to be at least as much influenced by Covid-19 as by the novel pandemic scenario and most people reported that they gave mostly the same answers regarding the scenario as they would have done regarding Covid-19. Thus, it is possible that the omnipresent topic of the Covid-19 pandemic overshadowed other related health topics, making it difficult to create a scenario that is independent of the previous Covid-19 experience.

Another explanation is that the manipulation did in fact not change people’s lay beliefs about the world in the correct way. In this study, the manipulation’s effect on people’s lay beliefs about the world was assessed by asking people to self-report their beliefs. Thus, this measure tapped into what people report about their beliefs under optimal conditions (i.e., when having the time and resources to deliberate about their beliefs). However, a recent paper [47] suggests that lay beliefs (also known as implicit beliefs) are multifaceted in nature. There might be a second layer to these beliefs, consisting of people’s automatic, immediate responses instead of solely their deliberate consideration of the items under optimal conditions. A recent study showed that an implicit measure of lay beliefs of intelligence was predictive of people’s learning behavior [47]. Notably, these more automatic expressions of beliefs were shown to be a better predictor of behavior compared to people’s explicitly self-reported beliefs. It is possible that the short persuasive article only changed people’s more superficial, explicit beliefs, while their more automatic beliefs remained unchanged. Future studies could measure these automatic beliefs and test a manipulation that is tailored to changing this more automatic layer of lay beliefs about the world.

Finally, the generalizability of the sample needs to be addressed. The samples of all studies consisted of US Americans and specifically Amazon MTurk workers. Since Covid-19 and similar pandemics concern the whole world, it is vital to investigate processes underlying people’s perceptions regarding the pandemic in diverse samples. Lay beliefs about the world have been shown to differ between cultures [48,49]. Thus, their relationship to pandemic-related beliefs may also differ between countries. Future research should address this by investigating the association between lay beliefs about the world and pandemic-related perceptions in more diverse samples.

While we used the Health Belief Model as a guiding framework, it is important to note that we did not collect direct measures of all components of the model, but only perceived severity and perceived benefits. As a result, we cannot estimate the composite effect of all components, nor test interactions among Health Belief Model components. These choices were made to keep the study material concise and focus on the most relevant aspects to the research question, namely the relationship between lay beliefs about the world and pandemic-related outcomes. This called for a focus on severity and benefits in the pandemic context from a collective level perspective as reflected in the preregistration. Future research could address this gap by assessing all components of the Health Belief Model.

### Practical implications

Across six studies, we consistently found that lay beliefs about the world are linked to people’s perceptions of a pandemic threat, perceived benefits of protective behavior, and behavior intention. Importantly, however, our experimental manipulations of lay beliefs about the world were not successful in changing these perceptions and intentions. This may be because the underlying causality is more complex or because lay beliefs about the world are not fundamentally shifted by the short-term manipulations we employed. In any case, the underlying causality of this relationship is still unclear at this point. This limits the direct applicability of our findings for designing behavior-change strategies. Still, the results open two possible avenues for future research. First, future research may test stronger, more long-term interventions that may more effectively shift lay beliefs about the world. Such interventions may be more viable to change pandemic-related perceptions and intention and, thus, elucidate the underlying process more clearly. Second, even if lay beliefs about the world cannot easily be changed, they may serve as a marker to identify individuals or groups who are more likely to downplay threats and resist protective measures in the face of a pandemic. Since the respective scale is short, context-independent, and unobtrusive, it could be employed as a screening tool for targeted interventions (such as informational campaigns [50] or personal messages [51]). In this way, our findings inform both the limits and the opportunities of leveraging lay beliefs about the world to foster protective behavior in future pandemics.

## Conclusion

The global Covid-19 pandemic has put humanity into a situation where the protective behavior of each individual is needed. At the same time, people might not personally feel particularly threatened by the disease or they might think that their behavior will not make much of a difference. We explored how people’s lay beliefs about the changeability of the world influence these perceptions of the seriousness of the disease and the efficacy of protective behaviors and, as a result, people’s protective behavior. Although we find consistent correlations between believing in a changeable world and threat and efficacy beliefs and protective health behavior and intentions, we did not find support for a causal effect. We also tested a possible reversed causal effect of behavior on lay beliefs about the world but did not find evidence for this effect either. Overall, this indicates that the relationship between lay beliefs about the world and pandemic-related outcomes might be less straightforward. Besides, the results of a network analysis highlighted the importance of beliefs about the efficacy of health behaviors to protect others and alleviate the course of the health threat for behavioral intentions. Within a Health Belief Model framing, our data only speak to perceived severity and perceived benefits at the collective level; other Health Belief Model components were not measured and remain targets for future research. Clarifying when and for whom collective-referent benefits motivate protective behavior can be central for preparing for future pandemics. This provides interesting new insights on what motivates people’s health behavior in a global health threat.

## Supporting information

S1 File Supplement. (DOCX)
