# Supplementary material for: The relationship between lay beliefs about the world and pandemic-related beliefs, attitudes, and behaviors
Source: PLoS One. 2025 Dec 10;20(12):e0338367. doi: 10.1371/journal.pone.0338367 (PMC12694858; doi:10.1371/journal.pone.0338367)
Supplement: S1 File — (DOCX) [file pone.0338367.s001.docx]

# Supplement

## Study 2: additional measures on support of vaccination policy

Participants read a short description of a recent proposal by the WHO to mitigate the pandemic (namely, the necessity of providing 11 billion Covid-19 vaccine doses to poorer countries). We further informed participants that the US had already announced a policy to provide 500 million vaccine doses, but the country could further support this case. Subsequently, participants answered two questions. First, participants indicated how much additional money the US should provide to support vaccination in poorer countries in their opinion (using a slider from $0 to $50 billion). Second, we asked participants if they were eligible to pay taxes. If they affirmed this, they indicated how much of a tax increase they would be willing to pay to fund these expenditures (using a slider from 0% to 10% with an additional response option of more than 10%). The incremental and entity beliefs groups did not differ in their responses to any of the two items (see Table S1).

Table S1. Tests of Differences Between the Experimental Groups Regarding Support of Vaccination Policy in Study 2.

| **Outcome variable** | ***M* (*SD*)** | | ***df*** | **Statistic** | ***p*** | ***d*** |
| --- | --- | --- | --- | --- | --- | --- |
|  | **Incremental** | **Entity** |  | **(*t* or χ²)** |  |  |
| Supported governmental spending on vaccination | 10.63 (12.12) | 11.38 (13.32) | 395 | -0.59 | .555 | 0.06 |
| Tax increase willingness^1^ | 1.37 (1.85) | 1.44 (2.00) | 388 | -0.35 | .726 | 0.04 |

N = 397. t-tests were conducted unless otherwise indicated. ^1^N = 390. Participants that did not currently pay taxes did not answer this question.

## Studies 3a-c: full scenario text

**[Part 1]** Scientists in Brazil have identified a novel virus, the so-called SciX virus. First, the virus does not get much attention, but after a few weeks, it turns out to be very infectious. It spreads to several other countries, and a Public Health Emergency of International Concern is declared. As the virus spreads to all countries around the world, reliable experts state that a global pandemic is emerging.

You follow the information of several experts that you deem knowledgeable and reliable concerning this situation. This is how they describe the virus: The symptoms of the SciX virus can range from almost none to life-threatening. A mild course of the disease lasts for a few days, causing headaches, coughs and skin rashes. A more serious course of the disease can affect the veins, potentially blocking blood supply to the heart and causing heart attacks. In the worst case, the virus can result in hospitalization and even death. The experts have noticed that people differ in their susceptibility to the SciX virus depending on factors such as demographics. You are personally counted among the low susceptibility group. This means that it’s likely that you would suffer from only a mild course of disease if you got infected with the SciX virus. Other people, however, have a much larger risk of suffering from a serious course of disease, including hospitalization and death.


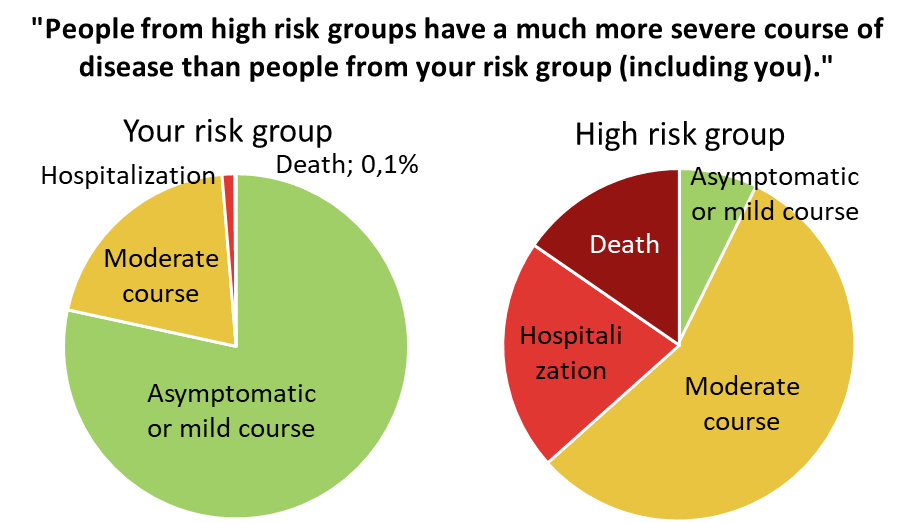


Transmission of the SciX virus occurs through physical contact, either by touching people that are infected by the virus themselves or by touching surfaces that are contaminated by the virus. People remain contagious for up to 20 days, and can spread the virus even if they do not develop symptoms. On surfaces, the virus can stay active for several days, depending on the specific surface. The risk of getting infected is highest when directly touching infected people and when touching surfaces that are newly contaminated and haven’t been properly cleaned.

Due to these facts, governments all over the world are implementing preventive measures to slow down the spread of the SciX virus and protect the people that are most vulnerable to the virus. International scientists that you consider experts on infectious diseases are issuing recommendations of personal behavior to reduce the risk of infections (most importantly social distancing).

**[Part 2]** Shortly after the SciX virus is discovered, scientists all over the world start researching possible vaccines against the SciX virus. Only a few months later, a Swiss biotechnology corporation starts testing a vaccine (called Scimed) in cooperation with a US-based pharmaceutical company. Scimed is tested over seven months with over 50,000 participants in several countries.

The vaccine is thoroughly discussed in the media and you follow the assessment of the experts that you consider most competent. According to them, the clinical trials show that two doses of Scimed can prevent symptoms from the SciX virus with high efficacy (approx. 95%). That means that participants who had been vaccinated had a 95% lower risk of getting symptoms from the SciX virus than participants who had not been vaccinated and had a 97% lower risk of passing the virus to others. Side-effects of the vaccine are limited to short-term side-effects. 70% of the participants experienced strong headaches, chills and exhaustion over 3-4 days after the vaccination. 10% experienced fever and flu-like symptoms that lasted up to a week. No long-term side-effects were observed. Due to the high efficacy and only short-term side-effects, Scimed is starting to get approved by the national drug regulation authorities. France is the first country to approve it, and countries all over the world follow shortly after.


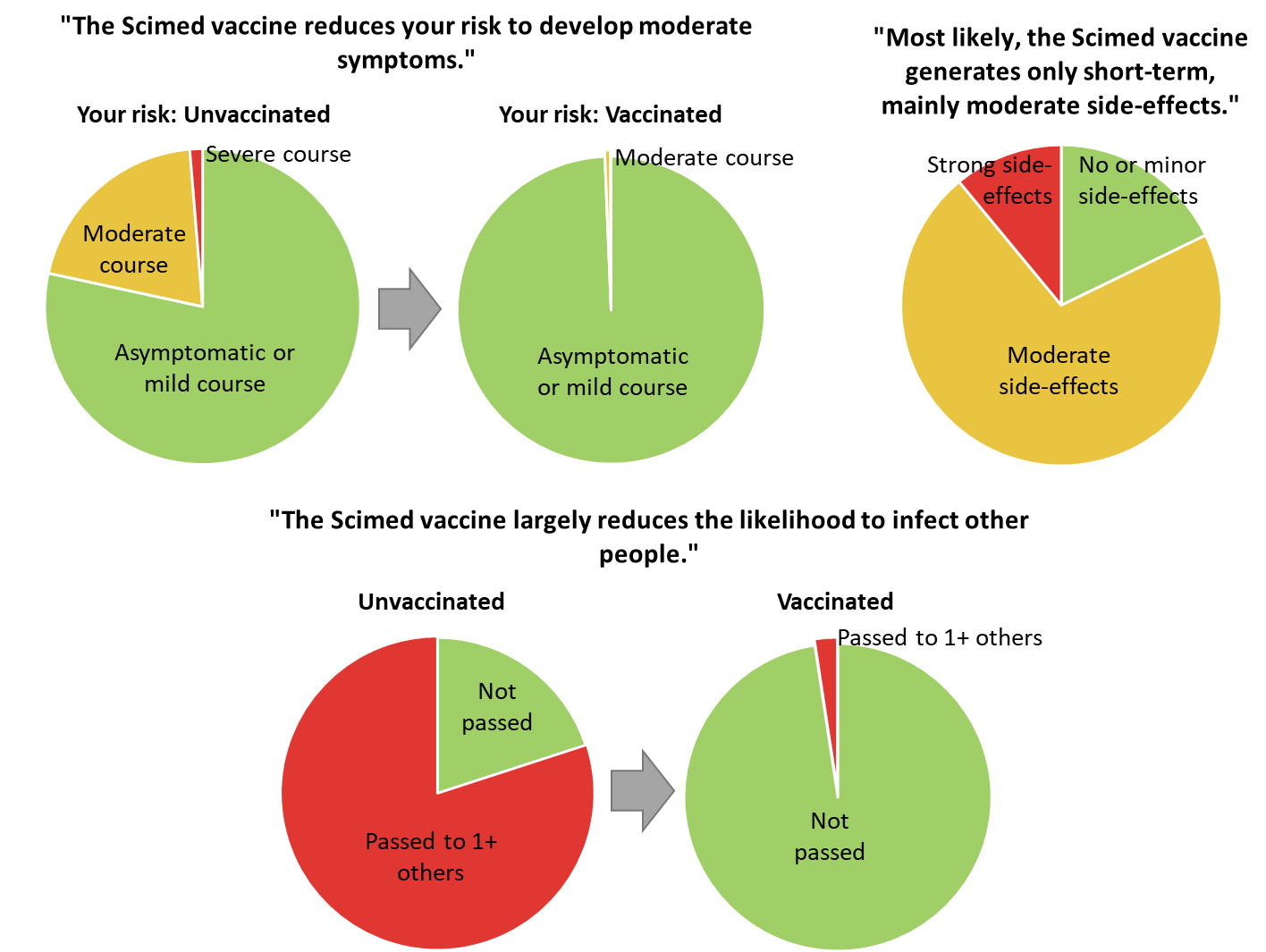


First, the supply of Scimed is still limited, and the most vulnerable people are prioritized to get vaccinated first. But soon after, the vaccine becomes available to anyone as well as to you.

National governments encourage their citizens to get vaccinated. They emphasize that getting vaccinated does not only protect oneself from the SciX virus, but also helps protect other people. Getting vaccinated reduces the chance of passing the SciX virus to other people.

## Studies 3a-c: descriptive results of the control questions

Table S2. Descriptive Results of the Control Questions on the SciX Virus Scenario in Studies 3a-c.

| **Question** | **1** | **2** | **3** | **4** | **5** |
| --- | --- | --- | --- | --- | --- |
| 1. *When reading the scenario about the SciX virus and answering the questions, to what extent did you think of Covid-19? (1 = not at all; 5 = completely)* | | | | | |
| Study 3a | 3% | 12% | 31% | 38% | 16% |
| 1. *To what extent did your experience with Covid-19 influence your answers on the questions? (1 = not at all; 5 = completely)* | | | | | |
| Study 3a | 9% | 17% | 29% | 31% | 13% |
| 1. *Which of the two rather influenced your answers to the questions on the SciX virus scenario? (1 = the information you read about the SciX virus; 5 = your views about Covid-19)* | | | | | |
| Study 3a | 19% | 23% | 36% | 13% | 8% |
| Study 3b | 24% | 21% | 36% | 12% | 7% |
| Study 3c | 23% | 16% | 45% | 9% | 7% |
| 1. *Were your answers similar to what you would have answered to questions about Covid-19? (1= very different answers; 5 = exactly the same answers)* | | | | | |
| Study 3a | 0% | 2% | 7% | 63% | 27% |
| Study 3b | 1% | 4% | 14% | 62% | 19% |
| Study 3c | 1% | 2% | 16% | 56% | 25% |
| 1. *Comparing the described SciX virus to the current coronavirus (SARS-CoV-2), what would you say: Symptoms from the SciX virus are... (1 = milder; 5 = more serious)* | | | | | |
| Study 3a | 3% | 14% | 62% | 17% | 4% |
| Study 3b | 3% | 13% | 61% | 19% | 4% |
| 1. *The SciX virus requires... (1 = softer measures; 5 = stricter measures)* | | | | | |
| Study 3a | 2% | 10% | 63% | 18% | 7% |
| Study 3b | 1% | 8% | 52% | 27% | 12% |
| 1. *Comparing the described Scimed vaccine to the BionTech vaccine against Covid-19, what would you say: Scimed is... (1 = less effective in preventing disease; 5 = more effective in preventing disease)* | | | | | |
| Study 3a | 0% | 3% | 62% | 24% | 12% |
| Study 3b | 4% | 16% | 61% | 13% | 6% |
| 1. *The side-effects from Scimed are... (1= milder; 5 = more serious)* | | | | | |
| Study 3a | 2% | 8% | 62% | 23% | 5% |
| Study 3b | 3% | 7% | 50% | 32% | 9% |

Percentage of participants that chose the respective response options on the control questions in Studies 3a-c.

## Study 3a: results when controlling for age

Table S3. Multiple Linear Regressions of the Effect of the Experimental Group on the Pandemic-Related Outcomes While Controlling for Age.

| **Outcome** | ***b*** | ***CI*** | ***t*** | ***p*** |
| --- | --- | --- | --- | --- |
| *Vaccination intention* |  |  |  |  |
| (Intercept) | 4.42 | 4.02 – 4.82 | 21.54 | <.001*** |
| Group | -0.13 | -0.40 – 0.15 | -0.89 | .373 |
| Age | -0.00 | -0.01 – 0.01 | -0.20 | .841 |
| *Vaccination readiness* |  |  |  |  |
| (Intercept) | 4.35 | 4.04 – 4.66 | 27.67 | <.001*** |
| Group | -0.07 | -0.29 – 0.14 | -0.68 | .495 |
| Age | 0.00 | -0.01 – 0.01 | 0.27 | .785 |
| *Health threat-related behavior change* |  |  |  |  |
| (Intercept) | 3.98 | 3.72 – 4.24 | 30.55 | <.001*** |
| Group | 0.09 | -0.09 – 0.26 | 0.99 | .325 |
| Age | 0.00 | -0.00 – 0.01 | 1.28 | .202 |
| *Threat belief* |  |  |  |  |
| (Intercept) | 4.89 | 4.58 – 5.20 | 31.28 | <.001*** |
| Group | -0.02 | -0.23 – 0.20 | -0.14 | .888 |
| Age | 0.00 | -0.01 – 0.01 | 0.50 | .616 |
| *Efficacy beliefs about mitigation efforts* |  |  |  |  |
| (Intercept) | 4.55 | 4.21 – 4.88 | 26.51 | <.001*** |
| Group | -0.11 | -0.35 – 0.12 | -0.97 | .334 |
| Age | 0.00 | -0.00 – 0.01 | 1.03 | .304 |
| *Efficacy beliefs about individual mitigation efforts* | |  |  |  |
| (Intercept) | 4.46 | 4.09 – 4.83 | 23.84 | <.001*** |
| Group | -0.24 | -0.49 – 0.01 | -1.89 | .060† |
| Age | 0.00 | -0.01 – 0.01 | 0.84 | .404 |
| *Efficacy beliefs about vaccination* |  |  |  |  |
| (Intercept) | 4.77 | 4.38 – 5.16 | 24.04 | <.001*** |
| Group | -0.18 | -0.45 – 0.08 | -1.35 | .179 |
| Age | 0.01 | -0.00 – 0.02 | 1.11 | .270 |
| *Efficacy beliefs about individual vaccination* |  |  |  |  |
| (Intercept) | 4.62 | 4.19 – 5.04 | 21.20 | <.001*** |
| Group | -0.29 | -0.59 – -0.00 | -1.97 | .050* |
| Age | 0.01 | -0.00 – 0.02 | 1.43 | .153 |
| *Attitudes towards measures* |  |  |  |  |
| (Intercept) | 4.28 | 3.87 – 4.69 | 20.60 | <.001*** |
| Group | 0.05 | -0.23 – 0.33 | 0.33 | .740 |
| Age | -0.02 | -0.03 – -0.01 | -3.03 | .003** |

N = 271. Experimental group is coded as 0 = incremental belief group, 1 = entity belief group. † p < .10, * p < .05, ** p < .01, *** p < .001
